# Racial/Ethnic Disparities in Neoplasm-Related Mortality and the Social Determinants of Health

**DOI:** 10.3390/cancers18101572

**Published:** 2026-05-12

**Authors:** Yoshito Nishimura, Mariko Fujii, Nanami Sako, Quynh Thi Vu, Ko Harada, Hideharu Hagiya, Urshila Durani, Stephen M. Ansell, James R. Cerhan, Toshihiro Koyama

**Affiliations:** 1 Division of Hematology, Mayo Clinic, Rochester, MN 55905, USA; 2 Department of Health Data Science, Graduate School of Medicine, Dentistry and Pharmaceutical Sciences, Okayama University, Okayama 700-8558, Japan; 3 Brookdale Department of Geriatrics and Palliative Medicine, Icahn School of Medicine at Mount Sinai, New York, NY 10003, USA; 4 Department of Infectious Diseases, Okayama University Hospital, Okayama 700-8558, Japan; 5 Department of Quantitative Health Sciences, Mayo Clinic, Rochester, MN 55905, USA

**Keywords:** healthcare disparities, disease, neoplasms/mortality, regression analysis, preventive health services, mortality, trends

## Abstract

**Background/Objectives**: Racial/ethnic and regional disparities in neoplasm-related mortality remain a significant public health challenge. In this study, we aimed to evaluate long-term trends in county-level neoplasm-related mortality rates by race/ethnicity in the United States and examine associations with social determinants of health. **Methods**: We conducted a cross-sectional ecological study using population-based data from the Global Burden of Disease Study, including individuals residing in 50 states of the United States and the District of Columbia from 2000 to 2019. We analyzed age-standardized neoplasm-related mortality rates by ethnicity/race. Joinpoint regression analysis was used to identify significant changes in mortality trends, summarized as average annual percentage change. County-level correlations between mortality and key social determinants of health were also assessed. **Results**: Neoplasm-related mortality rates declined across all racial/ethnic groups from 2000 to 2019; however, disparities persisted. The age-standardized neoplasm-related mortality rates per 100,000 population decreased in all racial/ethnic subgroups. The average annual percentage change ranged from −0.94% (Hispanic and non-Hispanic American Indian or Alaska Native) to −1.90% (Black). Sex-specific analyses revealed similar trends. Southeastern states experienced slower declines than Northeastern states did. County-level smoking and poverty rates were positively correlated, whereas the primary care physician-to-population ratio, excessive alcohol consumption rate, mammography screening rate, and median household income were inversely correlated with neoplasm-related mortality rate, varying by race/ethnicity. **Conclusions**: Targeted, community-specific interventions are required to reduce inequities in cancer outcomes.

## Simple Summary

Cancer mortality rates in the United States differ significantly across racial/ethnic groups and geographic regions, yet few studies have examined these disparities at the county level alongside social determinants of health. Using Global Burden of Disease data, we analyzed age-standardized neoplasm-related mortality rates across all US states and counties, stratified by five racial/ethnic groups. Although mortality declined in all groups, substantial disparities persisted, with non-Hispanic Black and non-Hispanic American Indian or Alaska Native populations experiencing the highest rates. Southeastern states showed slower improvements than Northeastern states. At the county level, higher smoking and poverty rates were associated with increased cancer mortality, while greater primary care access, mammography screening, and household income were associated with lower mortality, with associations varying by race/ethnicity. These findings highlight the need for geographically tailored and culturally responsive interventions addressing modifiable social determinants to reduce persistent cancer inequities.

## 1. Introduction

Cancer is one of the leading causes of death in the United States (US), with over 600,000 deaths attributed to neoplastic diseases annually [1,2,3]. Despite overall improvements in cancer-related mortality rates in recent decades, persistent disparities across racial and ethnic groups continue to pose significant public health challenges [4]. The non-Hispanic (NH) Black, NH American Indian or Alaska Native (AIAN), and Latino populations experience disproportionately higher cancer-related mortality rates than NH White and Asian/Pacific Islanders (API) do, and this is often linked to inequalities in socioeconomic status, healthcare access, and environmental exposures [5,6,7]. Moreover, these disparities are inconsistent across the country—they vary substantially by state and region, which highlights the importance of geographically detailed analyses [8,9]. Understanding how neoplasm-related mortality trends evolve within specific racial/ethnic subgroups and across states is critical for developing equitable targeted cancer control strategies.

Although previous studies have documented racial/ethnic disparities in cancer outcomes at national and regional levels [10,11,12,13], few have systematically examined long-term trends in neoplasm-related mortality disaggregated by race/ethnicity at the state and county levels in relation to social determinants of health (SDOH). Furthermore, evidence regarding how these trends correspond to modifiable structural factors, such as healthcare access metrics, socioeconomic status, and preventive measures, is limited [14,15,16]. This lack of granular population-based analyses limits opportunities for implementing precision public health approaches. The temporal dynamics of cancer-related mortality, particularly in minority populations, such as the NH AIAN and Latino communities, remain poorly characterized across US states. The lack of detailed data limits policymakers’ ability to design state- and subgroup-specific interventions to reduce avoidable cancer-related deaths.

To address this critical gap, we utilized data from the Global Burden of Disease (GBD) study, aiming to examine age-standardized neoplasm-related mortality rates by race/ethnicity across all 50 states and the District of Columbia (DC) from 2000 to 2019, along with county-level socioeconomic, cancer-preventive, and healthcare-access indicators.

## 2. Materials and Methods

The methodological framework used to estimate neoplasm-related mortality rates by racial/ethnic group in this study was adopted from a previous GBD study in which the authors estimated cause-specific mortality rates by county and racial/ethnic group [17,18].

### 2.1. Data Sources

The Global Health Data Exchange of the Institute of Health Metrics and Evaluation (IHME) was used to obtain annual data on age-standardized neoplasm-related mortality rates in the US at the national, state, and county levels by racial/ethnic group and sex from 2000 to 2019. We restricted the analysis to 2000 to 2019 to evaluate pre-pandemic trends and avoid conflating underlying long-term disparities with the substantial cancer care disruptions during the COVID-19 era. Underlying causes of death were classified based on the International Classification of Diseases, 10th Revision (ICD-10) codes to GBD causes [19]. The IHME generated these estimates using population and death data from the National Center for Health Statistics. The neoplasm-related mortality rates by racial/ethnic group were determined using comparison charts and maps. Additional data pertaining to socioeconomic status were obtained from the Small-Area Income and Poverty Estimates program of the US Census Bureau.

Information on healthcare access and metrics on cancer prevention measures were derived from the County Health Rankings & Roadmaps program developed by the University of Wisconsin Population Health Institute [20,21,22,23].

### 2.2. Study Population and Definitions

The study population included all individuals who resided in the 50 US states and the DC from 2000 to 2019. The following mutually exclusive racial/ethnic categories were used: non-Latino and NH AIAN (AIAN), non-Latino and NH API (API), non-Latino and NH Black (Black), Latino or Hispanic (Latino) of any race, and non-Latino and NH White (White). Neoplasm-related mortality was defined as death primarily attributed to malignant, in situ, benign, and unspecified; or other neoplasms (ICD-10: C00–96; D00–49; K62 and 63; N60, 84, and 87), consistent with the GBD standards (Table S1). We used age-standardized mortality rates (ASRs) for each racial/ethnic group, with separate analyses conducted for male and female individuals.

### 2.3. Mortality Rate Estimation

In the GBD study, the cause-specific mortality rate was estimated using the Cause of Death Ensemble Model, a Bayesian meta-regression framework that incorporates covariates, study-level random effects, and ensemble modeling techniques to improve predictive accuracy. The input data sources included death certificates, cancer registries, and verbal autopsy studies, which were adjusted for completeness, misclassification, and garbage coding using established GBD algorithms. ASRs were computed per 100,000 population and standardized to the 2010 US Census population to enable comparisons across states, periods, and demographic subgroups [17].

The ASR per 100,000 population used herein is the sum of the products of age-specific ratios (a1) across categorized age groups (i) and the proportional weight (wi) of each age stratum (i) within the reference population, divided by the aggregate weights of all the age groups comprising this standardized population [24]. Estimates were extracted with 95% uncertainty intervals (UIs) derived from 1000 posterior draws to reflect data and model structure variability.

### 2.4. Trend Analysis

Temporal trends in the ASR of neoplasms by race/ethnicity from 2000 to 2019 at the county level were analyzed using joinpoint regression modeling with Joinpoint software, version 5.3.0 [25,26]. This statistical approach delineates changes in data trajectories by connecting multiple distinct line segments at defined “joinpoints” on a logarithmic scale. The US National Cancer Institute developed the Joinpoint software tool used herein to analyze data from the Surveillance, Epidemiology, and End Results (SEER) program and other epidemiological surveillance datasets. The annual percentage change (APC) was calculated for each identified time segment. Across the study period, the average annual percentage change (AAPC) was estimated as a summary measure of the overall trend. Trends were interpreted as increasing, decreasing, or stable based on the direction and statistical significance of the APC or AAPC.

### 2.5. Assessment of SDOH

To examine the potential association of SDOH—including socioeconomic status, healthcare access, and preventive metrics—with neoplasm-related mortality rates, we evaluated the ratio of the population to primary care physicians, median household income, total number of people living in poverty, mammography screening rates, excessive alcohol consumption rates, and adult smoking rates.

When SDOH datasets were available, we calculated the Spearman correlation coefficients between each variable and the ASRs for each racial/ethnic group at the county level until 2019. The coefficients and 95% confidence intervals are reported to describe the direction and magnitude of county-level associations, and were considered statistically significant at *p* < 0.05. Analyses were conducted on the data of the overall population.

### 2.6. Statistical Analyses

All statistical analyses were performed using R version 4.4.2 (R Foundation for Statistical Computing, Vienna, Austria). APCs, AAPCs, and their 95% confidence intervals (CIs) were derived using the Joinpoint Regression Program, version 5.3.0, with a significance level of α = 0.05. Maps and heat plots were generated to visualize the geographic variation in the compound annual growth rate (CAGR) of neoplasm-related mortality across the US [27].

## 3. Results

### 3.1. National Trends in Neoplasm-Related Mortality Rate by Race/Ethnicity (2000–2019)

**Figure 1:**
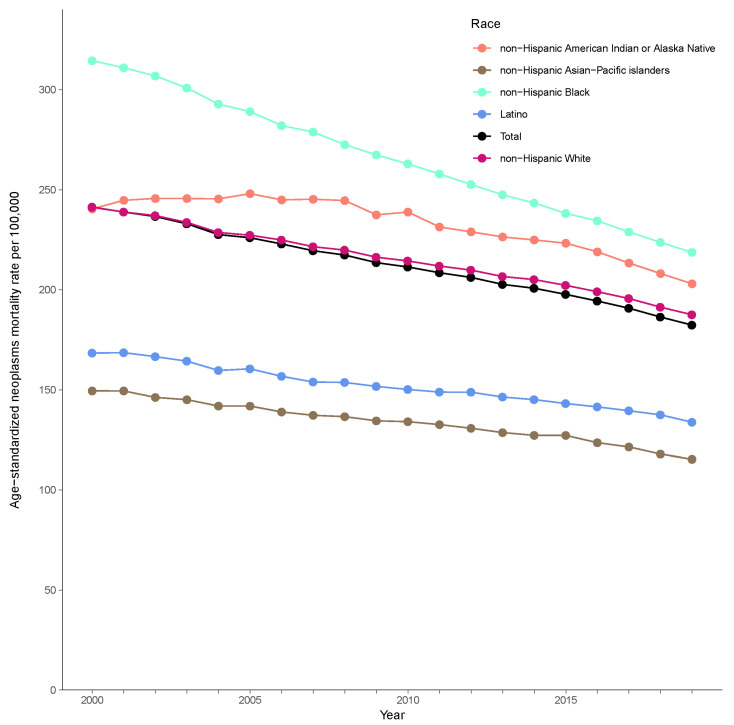
Age-standardized neoplasm-related mortality rates per 100,000 population by race/ethnicity in the United States, 2000–2019.

From 2000 to 2019, the ASRs for neoplasms decreased across all racial/ethnic groups in the US. In 2000, the highest ASR was observed among NH Black individuals (314.5 per 100,000; 95% UI: 312.1–317.0), followed by among NH White (241.2 per 100,000; 95% UI: 240.5–242.0), NH AIAN (240.5 per 100,000; 95% UI: 214.3–269.9), Latino (168.4 per 100,000; 95% UI: 164.6–172.0), and NH API (149.5 per 100,000; 95% UI: 144.9–153.9) individuals. By 2019, the ASRs had decreased substantially: 218.7 per 100,000 (95% UI: 217.1–220.3) for NH Black, 187.5 per 100,000 (95% UI: 186.9–188.2) for NH White, 203.0 per 100,000 (95% UI: 180.8–228.3) for NH AIAN, 133.8 per 100,000 (95% UI: 131.0–136.4) for Latino, and 115.3 per 100,000 (95% UI: 111.7–118.7) for NH API individuals (Figure 1, Table 1).

**Figure 2:**
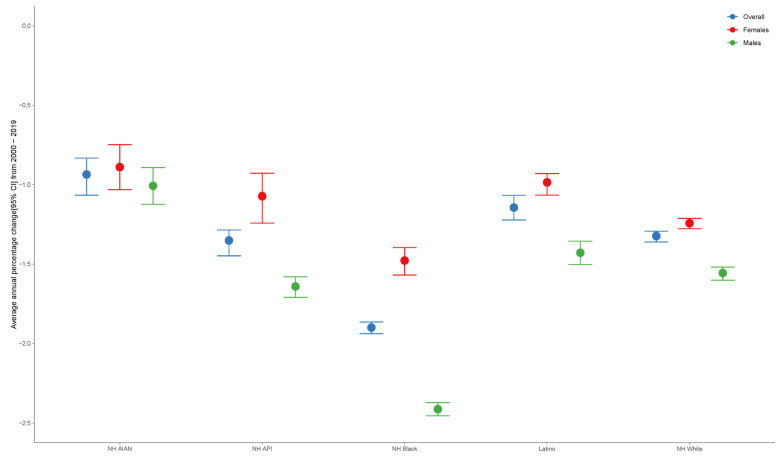
AAPC in neoplasm-related mortality rates from 2000 to 2019 by race/ethnicity and sex. Abbreviations: AAPC, average annual percentage change; CI, confidence interval; NH, non-Hispanic; NH AIAN, non-Hispanic American Indian or Alaska Native; NH API, non-Hispanic Asian-Pacific Islanders.

Joinpoint analysis revealed a significant decrease in the AAPC in ASR across all groups. The most considerable reduction was observed among NH Black individuals (AAPC: −1.90%; 95% CI: −1.94 to −1.86), followed by among NH API (−1.35%), Latino (−1.14%), NH White (−1.32%), and NH AIAN individuals (−0.94%) (Figure 2). Sex-specific analyses revealed more significant decreases in male individuals than in female individuals across most groups, although the relative order of disparities remained consistent (Figure S1). Overall, despite the overall mortality decline, there were persistent disparities, with NH Black and NH AIAN populations remaining disproportionately affected.

### 3.2. Geographic Variation in Neoplasm-Related Mortality Trends by State and County

**Figure 3:**
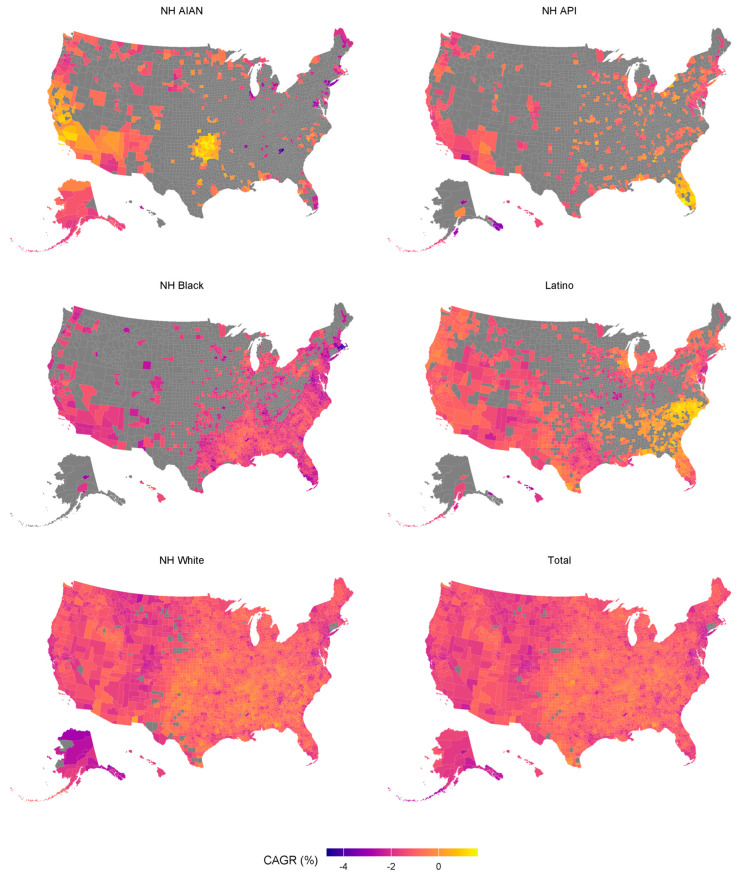
Choropleth map showing compound annual growth rates of neoplasm-related mortality by race/ethnicity across US states. Abbreviations: CAGR, compound annual growth rate; NH, non-Hispanic; NH AIAN, non-Hispanic American Indian or Alaska Native; NH API, non-Hispanic Asian-Pacific Islanders.

Although most states experienced an overall decrease in ASR, the pace of improvement varied significantly by region and racial/ethnic group (Figure S2). States in the Northeast and West, including New York, California, and Massachusetts, saw more rapid declines in ASR. In contrast, several Southeastern states, such as Mississippi, Alabama, and Kentucky, showed slower progress and, in some instances, stagnating or worsening trends. Although all racial/ethnic groups showed overall declines across 2000–2019, the joinpoint analyses indicated that the slope of decline was not constant over time at the state level, with some groups showing steeper reductions in later years. Table S2 summarizes US state-level trends in ASRs in different race/ethnicities, including joinpoint years and segmental APCs. For example, NH AIAN populations exhibited persistently high neoplasm-related mortality rates, with slow improvements in states such as South Dakota and Montana. Similarly, Latino populations in several Southern states, such as Texas and Arkansas, experienced slower declines or plateauing trends in comparison with the national averages. In contrast, the NH API population showed consistent and substantial reductions in mortality rate across nearly all states, with ASRs falling below 120 per 100,000 population by 2019 in most states. Figure 3 illustrates the county-level mapping of CAGR from 2000 to 2019 stratified by race/ethnicity. At the county level, different geographical trends were observed depending on race/ethnicity (Table S3). The highest ASRs were observed for NH AIAN and NH White populations in Northeastern, Midwestern, and Southwestern counties.

For the NH API population, counties in the Midwest or Southwest showed the highest ASRs. For the NH Black population, the highest ASRs were noted in the Southeastern and Southwestern counties. The highest ASRs in the Latino population were found in the Southwest and Hawaii. The findings indicate that neoplasm-related mortality trends have been geographically uneven, with several Southeastern states lagging behind other regions.

### 3.3. Correlation Between Neoplasm-Related Mortality Rate and Social Determinants of Health

The correlations between neoplasm-related mortality rate and socioeconomic status variables or preventive measures are summarized in Figure S3 and Table S4. Overall, the adult smoking rate was positively correlated with the neoplasm-related mortality rate at the county level in 2011 and 2019 (r = 0.642 and r = 0.728, respectively), with NH Black and NH White populations being disproportionately affected. Excessive alcohol consumption showed an inverse correlation with neoplasm-related mortality rate in NH Black and NH White populations. The mammography screening rate and primary care physician-to-population ratio showed an inverse correlation with neoplasm-related mortality rate, except for the mammography screening rate among the NH AIAN and NH API populations and the primary care physician-to-population rate among the Latino population. Socioeconomic status, including the median household income and poverty rate, was significantly correlated with neoplasm-related mortality rate, with sequential increases in the absolute correlation coefficient from 2000 to 2019, except in the Latino population. The NH Black and NH White populations were disproportionately affected by the measured socioeconomic status, compared with other races/ethnicities.

## 4. Discussion

This study provides new and policy-relevant insights into disparities in cancer-related mortality by coupling a geographic approach with an analysis of social determinants. To the best of our knowledge, this is the first comprehensive assessment of long-term trends in neoplasm-related mortality rates disaggregated by race/ethnicity across all US states and counties using the GBD 2019 dataset. In particular, the study extends prior literature in three ways. First, it characterizes 20-year neoplasm-related mortality trends across all US states and counties for major racial/ethnic groups. Second, it evaluates whether the geography of mortality changes differs across racial/ethnic populations. Third, it examines county-level associations with selected socioeconomic, healthcare-access, and preventive-health indicators to identify subgroup-specific ecological patterns that may inform public health strategies. Although the overall neoplasm-related mortality rate declined across all racial/ethnic groups between 2000 and 2019, significant disparities remained. In particular, the NH Black and NH AIAN populations continued to experience the highest mortality rates. Importantly, we observed marked geographic variations in the degree of the ecological correlation between SDOH and neoplasm-related mortality rates across different racial/ethnic groups. Our findings highlight the need for county-level patterns that may help prioritize more targeted subgroup-specific approaches to address the determinants of racial and regional disparities in cancer-related mortality. The practical implication of the findings is that cancer control efforts should not be designed as geographically uniform interventions. Counties with persistently high mortality among NH Black and NH AIAN populations, especially in regions with higher poverty and smoking burden, or reduced preventive-service access, may warrant more targeted outreach, screening, and healthcare-delivery strategies.

Methodologically, the GBD modeling framework has distinct advantages. Unlike traditional cancer surveillance data—such as SEER/National Program of Cancer Registries incidence and vital statistics of mortality, which may be limited or unstable for smaller populations and reported at the state or national level—the GBD approach incorporates multiple data sources and corrections for misclassification, which allows for stable mortality estimates for counties and racial/ethnic groups for which data are available. Notably, by incorporating SDOH measures, this study offers a novel perspective on disparities in US cancer-related mortality and contributes to a more actionable epidemiological landscape.

The persistence of racial/ethnic disparities in neoplasm-related mortality reflects the multifactorial and structural nature of inequities in cancer outcomes. NH Black individuals had the highest ASRs throughout the study period despite showing the most significant overall reduction in neoplasm-related mortality rate. These findings are consistent with those of prior national reports that highlight a disproportionately high cancer burden among Black Americans, likely driven by a combination of delayed diagnoses, poorer access to guideline-concordant care, socioeconomic deprivation, and structural racism within healthcare systems [28,29]. Similarly, the relatively slow progress in NH AIAN populations is particularly concerning, as it may reflect a longstanding underinvestment in healthcare infrastructure in tribal and rural communities [30,31]. Geographic isolation, chronic underfunding of the Indian Health Service, and culturally discordant care models have all been implicated in persistent cancer-related inequities in NH AIAN populations. In contrast, the NH API populations consistently showed the lowest mortality rates and fastest improvements, although this group was heterogeneous and high-burden subpopulations are often obscured within the aggregated estimates. Future studies should further disaggregate the NH AIAN and NH API populations (i.e., distinguishing American Indians from Alaska Natives and Pacific Islanders from Asians) to clarify the subgroups that have been the most disproportionately affected by malignancies.

Our study showed that Southeastern states experienced slower declines in neoplasm-related mortality than Northeastern and Western states did. These findings align with those of earlier studies showing the geographic clustering of high cancer-related mortality rates in areas with persistent poverty, low insurance coverage, and limited healthcare capacity [32,33]. For example, disproportionately high mortality rates are reported across multiple racial/ethnic groups in states such as Mississippi, Kentucky, and Alabama, which may reflect the disadvantages of socioeconomic disparities and limited access to basic healthcare and preventive services. In contrast, states such as New York and California have better baseline access to care and more rapid reductions in mortality rate, which highlights the potential role of healthcare infrastructure, Medicaid expansion, and targeted public health programs [34]. These patterns support the growing consensus that modifiable and policy-level factors strongly affect cancer-related mortality.

The ecological correlations among SDOH, preventive variables, and neoplasm-related mortality further support that non-biological factors play a significant role in cancer outcomes [35]. Many SDOH-related factors, such as smoking and alcohol consumption, influence cancer development with long latency periods. Therefore, the observed cross-sectional correlations should be interpreted with caution as they may not reflect contemporaneous relationships between exposure and mortality. Still, our results suggest that the predominant factors contributing to neoplasm-related mortality may differ across racial/ethnic groups. These findings are partly consistent with previous literature demonstrating that geographic proximity to care, availability of oncology services, and specialist density are associated with earlier diagnosis, treatment adherence, and survival [14]. Notably, the weak or absent correlations observed among Latino and NH API populations may reflect more complex underlying factors, such as mobility patterns, differences in healthcare utilization, or sociocultural barriers that are not fully captured by the metrics used alone. Of note, the inverse ecological correlation observed for excessive alcohol consumption in NH White and NH Black should be interpreted with caution, as it is unlikely to indicate a protective effect and may instead reflect residual confounding by county urbanicity or differential self-reporting of alcohol use.

A major strength of this study lies in its integration of epidemiological trend analysis with an exploration of associations between SDOH, preventive metrics, and mortality data; this offers a more comprehensive understanding of the factors that perpetuate cancer-related inequities. This study also has some limitations. First, although the GBD mortality estimates were adjusted for coding inconsistencies and underreporting, the accuracy of the modeled data remained dependent on the quality of the source inputs, particularly for underrepresented groups, such as the NH AIAN population. Second, the broad racial/ethnic categories used in this study may have masked important within-group heterogeneity, especially among the AIAN, API, and Latino populations. AIAN combines distinct populations with different geographies and contexts, and API combines heterogeneous Asian and Pacific Islanders. Third, the use of multiple socioeconomic status variables and preventive measures may not have captured other important dimensions, such as quality of care, insurance status, cultural competency, and access to additional preventive and palliative services. SDOH data were only available from 2010 onward, which limited our ability to temporally align these variables with mortality data spanning 2000–2019. Because the SDOH analyses were ecological and county-level, the observed associations should not be interpreted as causal or as reflecting individual-level exposure–outcome relationships. Accordingly, these findings are best interpreted as descriptive ecological patterns that may lead to future individual-level studies, ideally within longitudinal cohort designs. County-level mortality estimates were attributed to the county of residence at death, which may not correspond to the location of early life environmental exposures or the location where cancer care was delivered. Residential mobility and care-seeking migration, including movement from rural to urban areas for specialty treatment, may have introduced geographic misclassification. Fourth, we did not distinguish between cancer types; therefore, site-specific mortality patterns may follow different trajectories and warrant independent investigation. Future studies should expand on these findings by incorporating site-specific cancer data, evaluating access to screening and treatment, and integrating SDOH at the community level. Also, because the study period spanned until the COVID-19 pandemic, the findings should be interpreted as reflecting pre-pandemic disparities and may not fully capture the post-pandemic changes in screening, diagnosis, and treatment access. From a policy perspective, these results highlight the urgency of developing geographically tailored and culturally responsive cancer control strategies, particularly in underserved regions where mortality rates remain considerably high. Closing equity gaps may require sustained investment in public health infrastructure, culturally competent care models, and equitable access to early detection, interventions, and survivorship care.

## 5. Conclusions

In conclusion, despite notable national declines in neoplasm-related mortality rates over the past two decades, substantial racial/ethnic and geographic disparities have persisted across the US, with regional variations remaining pronounced. The observed association between SDOH or preventive metrics and mortality underscores the importance of addressing these modifiable factors in generating hypotheses for intervention priorities, highlighting the critical need for data-driven targeted interventions that are both culturally and geographically tailored.

## Figures and Tables

**Table 1:** Age-standardized neoplasm-related mortality rate by racial/ethnic group in 2000–2019 ^†^.

	Overall		Males		Females	
	2000	2019	2000	2019	2000	2019
NH AIAN	240.5	203.0	290.6	240.1	207.8	174.9
	(214.3–269.9)	(180.8–228.3)	(253.3–333.1)	(210.7–273.7)	(183.2–237.0)	(154.7–197.9)
NH API	149.5	115.3	187.6	136.5	121.3	99.6
	(144.9–153.9)	(111.7–118.7)	(181.0–193.7)	(131.6–141.5)	(116.5–125.6)	(95.8–103.1)
NH Black	314.5	218.7	427.2	269.4	247.8	187.0
	(312.1–317.0)	(217.1–220.3)	(423.2–431.5)	(266.8–272.1)	(245.3–250.3)	(185.4–188.7)
NH Latino	168.4	133.8	214.5	161.3	138.1	114.5
	(164.6–172.0)	(131.0–136.4)	(208.9–219.9)	(157.5–164.9)	(134.3–141.4)	(111.8–117.1)
NH White	241.2	187.5	298.6	222.6	204.1	161.1
	(240.5–242.0)	(186.9–188.2)	(297.5–299.7)	(221.8–223.4)	(203.3–204.9)	(160.4–161.8)

Abbreviations: AIAN, American Indian or Alaska Native; API, Asian and Pacific Islanders; NH, non-Hispanic; ^†^ Presented as deaths per 100,000 population (95% uncertainty interval).

## Data Availability

All data generated or analyzed during this study are included in this published article and its Supplementary Information Files.

## Supplementary Materials

The following supporting information can be downloaded at https://www.mdpi.com/article/10.3390/cancers18101572/s1, Table S1: Neoplasm-related ICD-10 codes included in the study; Table S2: US state-level trends in age-standardized neoplasm-related mortality rates from 2000 to 2019; Table S3: Top 10 counties with highest and lowest age-standardized neoplasm-related mortality rates for each race/ethnicity in 2019; Table S4: County-level correlation coefficients between socioeconomic status variables and age-standardized neoplasm-related mortality rates for each race/ethnicity; Figure S1: Trends in age-standardized neoplasm-related mortality rates by US state and racial/ethnic group, stratified by sex: (a) men, (b) women, and (c) combined; Figure S2: Absolute change in age-standardized neoplasm-related mortality rates between 2000 and 2019 by US state and racial/ethnic group; Figure S3: County-level correlation coefficients between preventative measures and age-standardized neoplasm-related mortality rates for each race/ethnicity.

## Abbreviations

The following abbreviations are used in this manuscript:

AAPC	Average annual percentage change
AIAN	American Indian or Alaska Native
APC	Annual percentage change
API	Asian/Pacific Islanders
ASRs	Age-standardized mortality rates
CAGR	Compound annual growth rate
DC	District of Columbia
GBD	Global Burden of Disease
IHME	Institute of Health Metrics and Evaluation
NH	non-Hispanic
SEER	Surveillance, Epidemiology, and End Results
