# Supplementary material for: Racial/Ethnic Disparities in Neoplasm-Related Mortality and the Social Determinants of Health
Source: Cancers (Basel). 2026 May 12;18(10):1572. doi: 10.3390/cancers18101572 (PMC13204127; doi:10.3390/cancers18101572)
Supplement: Supplementary file 1 [file cancers-18-01572-s001.zip › cancers-4280649-supplementary.pdf]

Supplementary Information

Racial/Ethnic Disparities in Neoplasm-related Mortality and the Social Determinants of Health

Yoshito Nishimura, Mariko Fujii, Nanami Sako, Vu Quynh, Ko Harada, Hideharu Hagiya, Urshila Durani, Stephen M Ansell, James R Cerhan, Toshihiro Koyama

Table of Contents

Table S1. Neoplasm-related ICD-10 codes included in the study

Table S2. US state-level trends in age-standardized neoplasm-related mortality rates from 2000 to 2019

Table S3. Top 10 counties with highest and lowest age-standardized neoplasm-related mortality rates for each race/ethnicity in 2019

Table S4. County-level correlation coefficients between socioeconomic status variables and age-standardized neoplasm-related mortality rates for each race/ethnicity

Figure S1. Trends in age-standardized neoplasm-related mortality rates by US state and racial/ethnic group, stratified by sex: (a) men, (b) women, and (c) combined

Figure S2. Absolute change in age-standardized neoplasm-related mortality rates between 2000 and 2019 by US state and racial/ethnic group

Figure S3. County-level correlation coefficients between preventative measures and age-standardized neoplasm-related mortality rates for each race/ethnicity

Table S1. Neoplasm-related ICD-10 codes included in the study.

Cause Level	Cause	ICD-10 Codes
2	Neoplasms	C00–C13.9, C15–C22.8, C23–C25.9, C30–C34.9, C37–C38.8, C40–C41.9, C43–C45.9, C47–C54.9, C56–C57.8, C60–C63.8, C64–C67.9, C68.0–C68.8, C69.0–C69.8, C70–C73.9, C75–C75.8, C81–C82.9, C83.0–C83.8, C84–C85.0, C85.2–C85.8, C86–C86.6, C88–C91.0, C91.2–C91.3, C91.6, C92–C92.6, C93–C93.1, C93.3, C93.8, C94–C94.5, C94.7–C96.9, D00.1–D00.2, D01.0–D01.3, D02.0–D02.3, D03–D06.9, D07.0–D07.2, D07.4–D07.5, D09.0, D09.2–D09.3, D09.8, D10.0–D10.7, D11–D12.9, D13.0–D13.7, D14.0–D14.3, D15–D16.9, D22–D24.9, D26.0–D27.9, D28.0–D28.1, D28.7, D29.0–D29.8, D30.0–D30.8, D31–D36, D36.1–D36.7, D37.1–D37.5, D38.0–D38.5, D39.1–D39.2, D39.8, D40.0–D40.8, D41.0–D41.8, D42–D43.9, D44.0–D44.8, D45–D47.9, D48.0–D48.6, D49.2–D49.4, D49.6, K62.0–K62.1, K63.5, N60–N60.9, N84.0–N84.1, N87–N87.9
3	Lip and oral cavity cancer	C00–C08.9, D10.0–D10.5, D11–D11.9
3	Nasopharyngeal cancer	C11–C11.9, D10.6
3	Other pharyngeal cancer	C09–C10.9, C12–C13.9, D10.7
3	Esophageal cancer	C15–C15.9, D00.1, D13.0
3	Stomach cancer	C16–C16.9, D00.2, D13.1, D37.1
3	Colon and rectal cancer	C18–C21.9, D01.0–D01.3, D12–D12.9, D37.3–D37.5
3	Liver cancer	C22–C22.8, D13.4
4	Hepatoblastoma	C22.2
3	Gallbladder and biliary tract cancer	C23–C24.9, D13.5
3	Pancreatic cancer	C25–C25.9, D13.6–D13.7
3	Laryngeal cancer	C32–C32.9, D02.0, D14.1, D38.0
3	Tracheal, bronchial, and lung cancer	C33–C34.9, D02.1–D02.3, D14.2–D14.3, D38.1
3	Malignant skin melanoma	C43–C43.9, D03–D03.9, D22–D23.9, D48.5
3	Non-melanoma skin cancer	C44–C44.9, D04–D04.9, D49.2

Table S1. Neoplasm-related ICD-10 codes included in the study (continued).

Cause Level	Cause	ICD-10 Codes
4	Non-melanoma skin cancer (squamous-cell carcinoma)	C44–C44.9, D04–D04.9, D49.2
3	Soft tissue and other extraosseous sarcomas	C49–C49.9
3	Malignant neoplasm of bone and articular cartilage	C40–C41.9
3	Breast cancer	C50–C50.9, D05–D05.9, D24–D24.9, D48.6, D49.3
3	Cervical cancer	C53–C53.9, D06–D06.9, D26.0
3	Uterine cancer	C54–C54.9, D07.0–D07.2, D26.1–D26.9
3	Ovarian cancer	C56–C56.9, D27–D27.9, D39.1
3	Prostate cancer	C61–C61.9, D07.5, D29.1, D40.0
3	Testicular cancer	C62–C62.9, D29.2–D29.8, D40.1–D40.8
3	Kidney cancer	C64–C65.9, D30.0–D30.1, D41.0–D41.1
3	Bladder cancer	C67–C67.9, D09.0, D30.3, D41.4–D41.8, D49.4
3	Brain and central nervous system cancer	C70–C72.9, C75.1–C75.3
3	Eye cancer	C69.0–C69.8
4	Retinoblastoma	C69.2
4	Other eye cancers	C69.0–C69.1, C69.3–C69.8
3	Neuroblastoma and other peripheral nervous cell tumors	C47–C47.9
3	Thyroid cancer	C73–C73.9, D09.3, D09.8, D34–D34.9, D44.0
3	Mesothelioma	C45–C45.9
3	Hodgkin lymphoma	C81–C81.9

Table S1. Neoplasm-related ICD-10 codes included in the study (continued).

Cause Level	Cause	ICD-10 Codes
3	Non-Hodgkin lymphoma	C82–C82.9, C83.0–C83.8, C84–C85.0, C85.2–C85.8, C86–C86.6, C96–C96.9
4	Burkitt lymphoma	C83.7
4	Other non-Hodgkin lymphoma	C82–C82.9, C83.0–C83.6, C83.8, C84–C85.0, C85.2–C85.8, C86–C86.6, C96–C96.9
3	Multiple myeloma	C88–C90.9
3	Leukemia	C91–C91.0, C91.2–C91.3, C91.6, C92–C92.6, C93–C93.1, C93.3, C93.8, C94–C94.5, C94.7–C95.9
4	Acute lymphoid leukemia	C91.0, C91.2–C91.3, C91.6
4	Acute myeloid leukemia	C92.0, C92.3–C92.6, C93.0, C94.0, C94.2, C94.4–C94.5
4	Chronic myeloid leukemia	C92.1–C92.2
4	Other leukemia	C93.1, C93.3, C93.8, C94.1, C94.3, C94.7–C94.8
3	Other malignant neoplasms	C17–C17.9, C30–C31.9, C37–C38.8, C48–C48.9, C4A, C51–C52.9, C57–C57.8, C60–C60.9, C63–C63.8, C66–C66.9, C68.0–C68.8, C75–C75.0, C75.4–C75.8, D07.4, D09.2, D13.2–D13.3, D14.0, D15–D16.9, D28.0–D28.1, D28.7, D29.0, D30.2, D30.4–D30.8, D31–D31.9, D35–D35.2, D35.5–D36, D36.1–D36.7, D37.2, D38.2–D38.5, D39.2, D39.8, D41.2–D41.3, D44.1–D44.8, D48.0–D48.4
3	Other neoplasms	D32–D33.9, D35.3–D35.4, D42–D43.9, D45–D47.9, D49.6, K62.0–K62.1, K63.5, N60–N60.9, N84.0–N84.1, N87–N87.9
4	Myelodysplastic, myeloproliferative, and other hematopoietic neoplasms	D45–D47.9
4	Other benign and in situ neoplasms	N60–N60.9

ICD-10, International Classification of Diseases, 10th Revision
Search date for codes: October 1, 2025.

Table S2. US state-level trends in age-standardized neoplasm-related mortality rates from 2000 to 2019.

States	Race/Ethnicity	Cohort	Trend	Lower Endpoint	Upper Endpoint	APC	Lower CI	Upper CI	P-Value
Alabama	NH AIAN	NH AIAN - 2 joinpoints	1	2000	2007	-0.5661	-1.0254	0.1459	0.084
	NH AIAN	NH AIAN - 2 joinpoints	2	2007	2019	-2.1153*	-2.4071	-1.9071	< 0.001
	NH API	NH API - 2 joinpoints	1	2000	2005	0.0227	-0.3942	1.2472	0.90
	NH API	NH API - 2 joinpoints	2	2005	2019	-0.5459*	-1.1814	-0.4457	0.0048
	NH Black	NH Black - 4 joinpoints	1	2000	2002	-0.3114	-0.7113	0.0426	0.083
	NH Black	NH Black - 4 joinpoints	2	2002	2007	-1.0088*	-1.2931	-0.9294	< 0.001
	NH Black	NH Black - 4 joinpoints	3	2007	2016	-1.841*	-1.8885	-1.7332	< 0.001
	NH Black	NH Black - 4 joinpoints	4	2016	2019	-2.2237*	-2.661	-2.0104	< 0.001
	Latino	Latino - 2 joinpoints	1	2000	2008	-0.6007*	-1.0959	-0.3294	< 0.001
	Latino	Latino - 2 joinpoints	2	2008	2019	0.5665*	0.3912	0.8237	< 0.001
	NH White	NH White - 3 joinpoints	1	2000	2007	-0.4676*	-0.5818	-0.2504	0.013
	NH White	NH White - 3 joinpoints	2	2007	2015	-0.9014*	-1.0463	-0.786	< 0.001
	NH White	NH White - 3 joinpoints	3	2015	2019	-1.7243*	-2.1998	-1.48	< 0.001
Alaska	NH AIAN	NH AIAN - 4 joinpoints	1	2000	2002	1.2526*	0.4043	1.9558	< 0.001
	NH AIAN	NH AIAN - 4 joinpoints	2	2002	2008	0.2201	-1.1615	0.3708	0.34
	NH AIAN	NH AIAN - 4 joinpoints	3	2008	2014	-1.0331*	-3.7591	-0.7926	< 0.0001
	NH AIAN	NH AIAN - 4 joinpoints	4	2014	2019	-4.0049*	-4.2932	-3.6629	< 0.0001
	NH API	NH API - 4 joinpoints	1	2000	2006	-2.9187*	-3.2585	-2.5851	< 0.001
	NH API	NH API - 4 joinpoints	2	2006	2012	2.3049*	1.9824	2.7347	0.016

	NH API	NH API - 4 joinpoints	3	2012	2015	-2.3932*	-3.1991	-0.1874	0.042
	NH API	NH API - 4 joinpoints	4	2015	2019	-4.8288*	-5.8492	-4.3419	< 0.001
	NH Black	NH Black - 3 joinpoints	1	2000	2007	-3.2392*	-3.473	-2.9966	< 0.001
	NH Black	NH Black - 3 joinpoints	2	2007	2013	1.7046*	1.4414	1.9608	< 0.001
	NH Black	NH Black - 3 joinpoints	3	2013	2019	-5.9925*	-6.2671	-5.7212	< 0.001
	Latino	Latino - 4 joinpoints	1	2000	2002	-1.6298*	-2.5157	-0.8189	< 0.001
	Latino	Latino - 4 joinpoints	2	2002	2007	-3.2062*	-3.8257	-2.9205	< 0.001
	Latino	Latino - 4 joinpoints	3	2007	2011	-1.4822*	-2.342	-0.6703	0.011
	Latino	Latino - 4 joinpoints	4	2011	2019	-0.2748	-0.4584	0.0656	0.075
	NH White	NH White - 3 joinpoints	1	2000	2007	-1.7078*	-1.875	-1.5909	< 0.001
	NH White	NH White - 3 joinpoints	2	2007	2013	-1.0054*	-1.1379	-0.8158	< 0.001
	NH White	NH White - 3 joinpoints	3	2013	2019	-3.2292*	-3.3507	-3.1131	< 0.001
Arizona	NH AIAN	NH AIAN - 2 joinpoints	1	2000	2017	-0.7167*	-0.8601	-0.0676	0.044
	NH AIAN	NH AIAN - 2 joinpoints	2	2017	2019	-1.7839*	-2.8587	-0.7171	< 0.001
	NH API	NH API - 3 joinpoints	1	2000	2007	-1.7733*	-2.0072	-1.5597	< 0.001
	NH API	NH API - 3 joinpoints	2	2007	2015	-0.027	-0.2018	0.4145	0.95
	NH API	NH API - 3 joinpoints	3	2015	2019	-0.8479*	-1.693	-0.4171	< 0.001
	NH Black	NH Black - 2 joinpoints	1	2000	2006	-2.4096*	-3.0217	-2.116	< 0.001
	NH Black	NH Black - 2 joinpoints	2	2006	2019	-1.399*	-1.5012	-1.2693	< 0.001
	Latino	Latino - 2 joinpoints	1	2000	2011	-2.1059*	-2.3015	-1.9454	< 0.0001
	Latino	Latino - 2 joinpoints	2	2011	2019	-0.5948*	-0.846	-0.2464	0.0072
	NH White	NH White - 4 joinpoints	1	2000	2006	-1.0108*	-1.1629	-0.808	< 0.001

	NH White	NH White - 4 joinpoints	2	2006	2013	-1.5277*	-1.8576	-1.394	< 0.001
	NH White	NH White - 4 joinpoints	3	2013	2016	-0.7604*	-1.1903	-0.5487	< 0.001
	NH White	NH White - 4 joinpoints	4	2016	2019	-1.5784*	-2.1066	-1.2599	< 0.001
Arkansas	NH AIAN	NH AIAN - 2 joinpoints	1	2000	2006	1.9174*	1.2473	2.7841	< 0.001
	NH AIAN	NH AIAN - 2 joinpoints	2	2006	2019	-1.4581*	-1.6932	-1.2409	< 0.001
	NH API	NH API - 3 joinpoints	1	2000	2007	-0.4811*	-1.1508	-0.2868	0.0032
	NH API	NH API - 3 joinpoints	2	2007	2015	0.0031	-0.1518	0.6197	0.76
	NH API	NH API - 3 joinpoints	3	2015	2019	-0.8811*	-1.7375	-0.4779	< 0.001
	NH Black	NH Black - 4 joinpoints	1	2000	2002	-0.3449*	-0.7249	-0.0277	0.030
	NH Black	NH Black - 4 joinpoints	2	2002	2007	-1.0046*	-1.2765	-0.9166	< 0.001
	NH Black	NH Black - 4 joinpoints	3	2007	2016	-1.7342*	-1.7848	-1.6577	< 0.001
	NH Black	NH Black - 4 joinpoints	4	2016	2019	-2.1698*	-2.5623	-1.9591	< 0.001
	Latino	Latino - 2 joinpoints	1	2000	2007	-0.8383*	-2.0761	-0.4512	0.0024
	Latino	Latino - 2 joinpoints	2	2007	2019	-0.0811	-0.2586	0.5841	0.61
	NH White	NH White - 4 joinpoints	1	2000	2006	-0.9225*	-1.2134	-0.7683	< 0.001
	NH White	NH White - 4 joinpoints	2	2006	2012	-0.2597	-0.4467	0.1312	0.15
	NH White	NH White - 4 joinpoints	3	2012	2015	-0.9998*	-1.5475	-0.4797	< 0.001
	NH White	NH White - 4 joinpoints	4	2015	2019	-2.1191*	-2.4989	-1.8925	< 0.001
California	NH AIAN	NH AIAN - 4 joinpoints	1	2000	2002	2.0345*	0.8271	3.2804	< 0.001
	NH AIAN	NH AIAN - 4 joinpoints	2	2002	2009	0.0059	-0.806	0.1914	0.72

	NH AIAN	NH AIAN - 4 joinpoints	3	2009	2015	0.6787*	0.365	1.3676	0.010
	NH AIAN	NH AIAN - 4 joinpoints	4	2015	2019	-2.0715*	-2.7294	-1.4668	< 0.001
	NH API	NH API - 2 joinpoints	1	2000	2015	-1.1635*	-1.2404	-1.0631	0.0024
	NH API	NH API - 2 joinpoints	2	2015	2019	-2.2531*	-3.2806	-1.7605	< 0.001
	NH Black	NH Black - 3 joinpoints	1	2000	2002	-0.9045*	-1.4206	-0.4309	< 0.001
	NH Black	NH Black - 3 joinpoints	2	2002	2016	-1.8814*	-1.9214	-1.8269	< 0.001
	NH Black	NH Black - 3 joinpoints	3	2016	2019	-2.2825*	-2.9199	-1.9996	< 0.001
	Latino	Latino - 1 joinpoint	1	2000	2019	-0.9319*	-0.9886	-0.8759	< 0.001
	NH White	NH White - 4 joinpoints	1	2000	2002	-1.3144*	-1.7436	-1.0178	< 0.001
	NH White	NH White - 4 joinpoints	2	2002	2005	-1.994*	-2.1342	-1.2795	< 0.001
	NH White	NH White - 4 joinpoints	3	2005	2015	-1.3256*	-1.3929	-1.2434	< 0.001
	NH White	NH White - 4 joinpoints	4	2015	2019	-2.1856*	-2.3471	-1.998	< 0.001
Colorado	NH AIAN	NH AIAN - 3 joinpoints	1	2000	2007	0.5927	-0.5727	1.3979	0.12
	NH AIAN	NH AIAN - 3 joinpoints	2	2007	2016	-1.3135	-1.5382	1.6001	0.19
	NH AIAN	NH AIAN - 3 joinpoints	3	2016	2019	-2.4121*	-4.4939	-1.4198	< 0.001
	NH API	NH API - 3 joinpoints	1	2000	2007	-1.9875*	-2.435	-1.7559	< 0.001
	NH API	NH API - 3 joinpoints	2	2007	2011	-0.325	-0.9176	0.377	0.31
	NH API	NH API - 3 joinpoints	3	2011	2019	-1.7151*	-2.0094	-1.5396	< 0.001
	NH Black	NH Black - 3 joinpoints	1	2000	2006	-2.2118*	-2.5091	-2.0206	< 0.001
	NH Black	NH Black - 3 joinpoints	2	2006	2012	-1.1917*	-1.4118	-0.7975	< 0.001
	NH Black	NH Black - 3 joinpoints	3	2012	2019	-2.5468*	-2.7179	-2.3874	< 0.001

	Latino	Latino - 1 joinpoint	1	2000	2019	-1.3264*	-1.3895	-1.2655	< 0.001
	NH White	NH White - 2 joinpoints	1	2000	2015	-1.5329*	-1.5666	-1.4969	< 0.001
	NH White	NH White - 2 joinpoints	2	2015	2019	-2.5021*	-2.776	-2.2527	< 0.001
Connecticut	NH AIAN	NH AIAN - 4 joinpoints	1	2000	2006	-0.5115*	-0.9587	-0.0444	0.036
	NH AIAN	NH AIAN - 4 joinpoints	2	2006	2012	-3.7845*	-4.5689	-3.3164	0.0068
	NH AIAN	NH AIAN - 4 joinpoints	3	2012	2015	-0.946*	-2.0201	-0.3206	0.0032
	NH AIAN	NH AIAN - 4 joinpoints	4	2015	2019	-3.3783*	-4.5268	-2.87	< 0.001
	NH API	NH API - 3 joinpoints	1	2000	2011	-0.3806*	-0.5158	-0.1888	0.030
	NH API	NH API - 3 joinpoints	2	2011	2015	-1.6697*	-2.1673	-0.4486	0.017
	NH API	NH API - 3 joinpoints	3	2015	2019	-2.9445*	-3.8281	-2.4739	< 0.001
	NH Black	NH Black - 4 joinpoints	1	2000	2002	-1.9971*	-2.4103	-1.6311	< 0.001
	NH Black	NH Black - 4 joinpoints	2	2002	2006	-2.9301*	-3.2229	-2.7441	< 0.001
	NH Black	NH Black - 4 joinpoints	3	2006	2011	-1.9525*	-2.0962	-1.6742	< 0.001
	NH Black	NH Black - 4 joinpoints	4	2011	2019	-2.2242*	-2.4093	-2.1595	< 0.001
	Latino	Latino - 2 joinpoints	1	2000	2015	-1.2078*	-1.3167	-1.0053	0.019
	Latino	Latino - 2 joinpoints	2	2015	2019	-2.4121*	-4.4846	-1.657	< 0.001
	NH White	NH White - 3 joinpoints	1	2000	2006	-1.3642*	-1.504	-1.0977	< 0.001
	NH White	NH White - 3 joinpoints	2	2006	2016	-1.8432*	-1.9226	-1.7243	< 0.001
	NH White	NH White - 3 joinpoints	3	2016	2019	-2.4304*	-3.0677	-2.0772	< 0.001
Delaware	NH AIAN	NH AIAN - 4 joinpoints	1	2000	2006	-1.004*	-1.4672	-0.383	0.018
	NH AIAN	NH AIAN - 4 joinpoints	2	2006	2012	-2.9497*	-4.1689	-2.4117	0.017

	NH AIAN	NH AIAN - 4 joinpoints	3	2012	2016	-0.8993	-1.725	0.2288	0.10
	NH AIAN	NH AIAN - 4 joinpoints	4	2016	2019	-3.1284*	-4.9784	-2.1459	< 0.001
	NH API	NH API - 3 joinpoints	1	2000	2004	-1.9336*	-3.2953	-1.1904	< 0.001
	NH API	NH API - 3 joinpoints	2	2004	2016	-0.8596	-0.9821	0.2927	0.11
	NH API	NH API - 3 joinpoints	3	2016	2019	-2.3881*	-4.6026	-1.3505	< 0.001
	NH Black	NH Black - 3 joinpoints	1	2000	2002	-1.7754*	-2.5217	-1.1207	< 0.001
	NH Black	NH Black - 3 joinpoints	2	2002	2006	-2.7172*	-3.141	-1.4522	< 0.001
	NH Black	NH Black - 3 joinpoints	3	2006	2019	-1.7972*	-2.1943	-1.5739	< 0.001
	Latino	Latino - 4 joinpoints	1	2000	2002	-1.4155*	-2.4781	-0.5014	0.0012
	Latino	Latino - 4 joinpoints	2	2002	2007	-2.7565*	-3.4974	-0.633	0.0040
	Latino	Latino - 4 joinpoints	3	2007	2014	-0.7595*	-1.4145	-0.2065	0.012
	Latino	Latino - 4 joinpoints	4	2014	2019	-1.8706*	-2.4488	-1.4682	< 0.001
	NH White	NH White - 2 joinpoints	1	2000	2006	-0.9661*	-1.2046	-0.4164	0.024
	NH White	NH White - 2 joinpoints	2	2006	2019	-1.5071*	-1.6479	-1.4252	< 0.001
District of Columbia	NH AIAN	NH AIAN - 4 joinpoints	1	2000	2002	-3.1322*	-4.642	-1.7915	< 0.001
	NH AIAN	NH AIAN - 4 joinpoints	2	2002	2012	-5.0251*	-5.7183	-4.8505	< 0.001
	NH AIAN	NH AIAN - 4 joinpoints	3	2012	2016	-0.0202	-0.7751	1.0326	0.88
	NH AIAN	NH AIAN - 4 joinpoints	4	2016	2019	-2.6154*	-4.3452	-1.6815	< 0.001
	NH API	NH API - 2 joinpoints	1	2000	2009	-2.6194*	-3.0347	-2.4003	< 0.001
	NH API	NH API - 2 joinpoints	2	2009	2019	-1.6772*	-1.8744	-1.3489	< 0.001
	NH Black	NH Black - 3 joinpoints	1	2000	2006	-1.9142*	-2.226	-1.7605	< 0.001
	NH Black	NH Black - 3 joinpoints	2	2006	2016	-1.5189*	-1.5957	-1.3708	< 0.001

	NH Black	NH Black - 3 joinpoints	3	2016	2019	-2.6858*	-3.191	-2.2891	< 0.001
	Latino	Latino - 3 joinpoints	1	2000	2006	2.8926*	2.5105	3.3501	< 0.001
	Latino	Latino - 3 joinpoints	2	2006	2013	0.5661*	0.2058	0.9666	0.0064
	Latino	Latino - 3 joinpoints	3	2013	2019	-1.4166*	-1.9306	-1.0422	< 0.001
	NH White	NH White - 3 joinpoints	1	2000	2006	-2.0476*	-2.3778	-1.7019	< 0.001
	NH White	NH White - 3 joinpoints	2	2006	2012	-4.5252*	-5.0893	-4.1666	< 0.001
	NH White	NH White - 3 joinpoints	3	2012	2019	-2.93*	-3.1766	-2.58	< 0.001
Florida	NH AIAN	NH AIAN - 3 joinpoints	1	2000	2005	0.3098	-0.1431	0.9955	0.17
	NH AIAN	NH AIAN - 3 joinpoints	2	2005	2016	-1.2297*	-1.3832	-1.0012	0.0056
	NH AIAN	NH AIAN - 3 joinpoints	3	2016	2019	-2.6094*	-4.0609	-1.804	< 0.001
	NH API	NH API - 2 joinpoints	1	2000	2006	1.9805*	1.5669	2.5021	< 0.001
	NH API	NH API - 2 joinpoints	2	2006	2019	-0.2664*	-0.4078	-0.1331	< 0.001
	NH Black	NH Black - 3 joinpoints	1	2000	2002	-1.4267*	-1.9976	-1.0398	< 0.001
	NH Black	NH Black - 3 joinpoints	2	2002	2013	-2.3333*	-2.4179	-2.2858	< 0.001
	NH Black	NH Black - 3 joinpoints	3	2013	2019	-0.9926*	-1.1263	-0.8601	< 0.001
	Latino	Latino - 3 joinpoints	1	2000	2002	-0.1134	-0.8877	0.535	0.56
	Latino	Latino - 3 joinpoints	2	2002	2010	-1.2441*	-1.6824	-1.1122	< 0.001
	Latino	Latino - 3 joinpoints	3	2010	2019	-0.749*	-0.8845	-0.5642	< 0.001
	NH White	NH White - 2 joinpoints	1	2000	2016	-1.0674*	-1.1004	-1.0278	< 0.001
	NH White	NH White - 2 joinpoints	2	2016	2019	-1.7212*	-2.3172	-1.3843	< 0.001
Georgia	NH AIAN	NH AIAN - 3 joinpoints	1	2000	2005	-0.8532	-1.6939	0.2919	0.11

	NH AIAN	NH AIAN - 3 joinpoints	2	2005	2012	-4.3733*	-6.0091	-3.8235	< 0.001
	NH AIAN	NH AIAN - 3 joinpoints	3	2012	2019	-2.6072*	-3.1458	-0.6773	0.022
	NH API	NH API - 2 joinpoints	1	2000	2015	-0.3028*	-0.3874	-0.1967	0.0016
	NH API	NH API - 2 joinpoints	2	2015	2019	-1.5024*	-2.4911	-0.9837	< 0.001
	NH Black	NH Black - 2 joinpoints	1	2000	2002	-1.1511*	-1.6742	-0.7994	< 0.001
	NH Black	NH Black - 2 joinpoints	2	2002	2019	-2.0414*	-2.0756	-2.0166	< 0.001
	Latino	Latino - 3 joinpoints	1	2000	2007	-1.0061*	-1.8632	-0.6996	0.0096
	Latino	Latino - 3 joinpoints	2	2007	2012	0.1168	-0.6434	0.9611	0.66
	Latino	Latino - 3 joinpoints	3	2012	2019	1.3748*	1.0576	2.2732	0.0040
	NH White	NH White - 2 joinpoints	1	2000	2015	-1.069*	-1.1103	-1.0258	< 0.001
	NH White	NH White - 2 joinpoints	2	2015	2019	-2.1002*	-2.4798	-1.8267	< 0.001
Hawaii	NH AIAN	NH AIAN - 2 joinpoints	1	2000	2016	-2.3447*	-2.4469	-2.192	< 0.001
	NH AIAN	NH AIAN - 2 joinpoints	2	2016	2019	-4.0643*	-5.7592	-2.9783	< 0.001
	NH API	NH API - 3 joinpoints	1	2000	2006	-0.56*	-0.7461	-0.3035	0.0036
	NH API	NH API - 3 joinpoints	2	2006	2016	-1.5325*	-1.6302	-1.4109	< 0.001
	NH API	NH API - 3 joinpoints	3	2016	2019	-2.4814*	-3.3578	-2.0367	< 0.001
	NH Black	NH Black - 3 joinpoints	1	2000	2002	0.5061	-0.4896	1.2912	0.34
	NH Black	NH Black - 3 joinpoints	2	2002	2007	-0.8109*	-2.5117	-0.6325	< 0.001
	NH Black	NH Black - 3 joinpoints	3	2007	2019	-2.3463*	-2.474	-2.1903	< 0.001
	Latino	Latino - 3 joinpoints	1	2000	2007	-3.6439*	-4.4194	-3.3588	< 0.001
	Latino	Latino - 3 joinpoints	2	2007	2012	-2.4731*	-3.0792	-0.9222	0.0080
	Latino	Latino - 3 joinpoints	3	2012	2019	-0.1417	-0.4507	0.4578	0.42

	NH White	NH White - 2 joinpoints	1	2000	2013	-1.2177*	-1.3024	-1.1172	< 0.001
	NH White	NH White - 2 joinpoints	2	2013	2019	-2.3114*	-2.654	-2.0433	< 0.001
Idaho	NH AIAN	NH AIAN - 4 joinpoints	1	2000	2007	-0.2821*	-0.519	-0.0206	0.040
	NH AIAN	NH AIAN - 4 joinpoints	2	2007	2012	-2.4423*	-3.0845	-2.0537	0.0092
	NH AIAN	NH AIAN - 4 joinpoints	3	2012	2016	-0.9321*	-1.3994	-0.2953	0.0016
	NH AIAN	NH AIAN - 4 joinpoints	4	2016	2019	-2.4595*	-3.4166	-1.9015	< 0.001
	NH API	NH API - 3 joinpoints	1	2000	2006	-1.1507*	-1.9046	-0.9053	< 0.001
	NH API	NH API - 3 joinpoints	2	2006	2015	-0.599	-0.728	0.0286	0.056
	NH API	NH API - 3 joinpoints	3	2015	2019	-1.6217*	-2.4231	-1.159	< 0.001
	NH Black	NH Black - 2 joinpoints	1	2000	2005	-2.6784*	-3.8006	-2.198	< 0.001
	NH Black	NH Black - 2 joinpoints	2	2005	2019	-1.8811*	-1.9838	-1.6168	< 0.001
	Latino	Latino - 2 joinpoints	1	2000	2007	-1.9324*	-2.3509	-1.7225	< 0.001
	Latino	Latino - 2 joinpoints	2	2007	2019	-1.3105*	-1.4164	-1.161	< 0.001
	NH White	NH White - 2 joinpoints	1	2000	2015	-0.8583*	-0.9011	-0.8079	< 0.001
	NH White	NH White - 2 joinpoints	2	2015	2019	-1.5204*	-2.0251	-1.254	< 0.0001
Illinois	NH AIAN	NH AIAN - 3 joinpoints	1	2000	2005	-0.7667*	-1.152	-0.2745	0.0048
	NH AIAN	NH AIAN - 3 joinpoints	2	2005	2009	-3.7061*	-4.2709	-3.1439	< 0.001
	NH AIAN	NH AIAN - 3 joinpoints	3	2009	2019	-2.8356*	-2.9656	-2.504	< 0.001
	NH API	NH API - 4 joinpoints	1	2000	2007	-1.4303*	-1.9114	-1.1972	0.0036
	NH API	NH API - 4 joinpoints	2	2007	2011	0.386	-0.1766	1.1282	0.13
	NH API	NH API - 4 joinpoints	3	2011	2015	-1.1949*	-1.8376	-0.7368	0.0056
	NH API	NH API - 4 joinpoints	4	2015	2019	-3.4105*	-4.0964	-2.9573	< 0.001

	NH Black	NH Black - 1 joinpoint	1	2000	2019	-1.903*	-1.9517	-1.8542	< 0.001
	Latino	Latino - 3 joinpoints	1	2000	2007	-1.3019*	-2.3331	-1.0102	0.0024
	Latino	Latino - 3 joinpoints	2	2007	2014	-0.4659	-0.7495	0.5291	0.27
	Latino	Latino - 3 joinpoints	3	2014	2019	-1.6074*	-2.6894	-1.192	0.0012
	NH White	NH White - 2 joinpoints	1	2000	2015	-1.2084*	-1.2467	-1.1669	< 0.001
	NH White	NH White - 2 joinpoints	2	2015	2019	-2.062*	-2.4578	-1.8081	< 0.001
Indiana	NH AIAN	NH AIAN - 3 joinpoints	1	2000	2005	0.2004	-1.6292	2.2021	0.60
	NH AIAN	NH AIAN - 3 joinpoints	2	2005	2017	-1.7123	-2.4012	1.3609	0.11
	NH AIAN	NH AIAN - 3 joinpoints	3	2017	2019	-3.4173*	-5.6755	-1.5367	< 0.001
	NH API	NH API - 4 joinpoints	1	2000	2007	-0.626*	-0.914	-0.4435	0.0044
	NH API	NH API - 4 joinpoints	2	2007	2011	1.1346*	0.7156	1.7658	0.017
	NH API	NH API - 4 joinpoints	3	2011	2015	-0.2066	-0.7462	0.1991	0.23
	NH API	NH API - 4 joinpoints	4	2015	2019	-2.0687*	-2.6509	-1.691	< 0.001
	NH Black	NH Black - 2 joinpoints	1	2000	2014	-1.7001*	-1.744	-1.638	< 0.001
	NH Black	NH Black - 2 joinpoints	2	2014	2019	-2.1646*	-2.5305	-1.969	< 0.001
	Latino	Latino - 3 joinpoints	1	2000	2007	-1.97*	-2.3543	-1.692	< 0.001
	Latino	Latino - 3 joinpoints	2	2007	2014	-0.0307	-0.2923	0.8661	0.90
	Latino	Latino - 3 joinpoints	3	2014	2019	-1.0253*	-1.8774	-0.6528	< 0.001
	NH White	NH White - 3 joinpoints	1	2000	2006	-1.1893*	-1.4441	-1.057	< 0.001
	NH White	NH White - 3 joinpoints	2	2006	2015	-0.705*	-0.7864	-0.5635	< 0.001
	NH White	NH White - 3 joinpoints	3	2015	2019	-1.6261*	-1.9215	-1.392	< 0.001
Iowa	NH AIAN	NH AIAN - 3 joinpoints	1	2000	2002	2.5669*	0.8021	3.9796	< 0.001

	NH AIAN	NH AIAN - 3 joinpoints	2	2002	2007	-0.1617	-1.4328	0.2824	0.32
	NH AIAN	NH AIAN - 3 joinpoints	3	2007	2019	-1.5311*	-1.7922	-1.3613	< 0.001
	NH API	NH API - 4 joinpoints	1	2000	2007	-1.4955*	-1.7385	-1.3377	< 0.001
	NH API	NH API - 4 joinpoints	2	2007	2011	1.0125*	0.5266	1.4765	0.046
	NH API	NH API - 4 joinpoints	3	2011	2015	-0.0386	-0.5207	0.4257	0.80
	NH API	NH API - 4 joinpoints	4	2015	2019	-1.5144*	-2.0576	-1.1888	< 0.001
	NH Black	NH Black - 4 joinpoints	1	2000	2002	-1.4892*	-1.9829	-1.1166	< 0.001
	NH Black	NH Black - 4 joinpoints	2	2002	2006	-2.261*	-2.5633	-1.0168	< 0.001
	NH Black	NH Black - 4 joinpoints	3	2006	2011	-0.9134*	-1.463	-0.7007	< 0.001
	NH Black	NH Black - 4 joinpoints	4	2011	2019	-1.7332*	-1.8212	-1.646	< 0.001
	Latino	Latino - 2 joinpoints	1	2000	2007	-2.4702*	-2.8744	-2.1826	< 0.001
	Latino	Latino - 2 joinpoints	2	2007	2019	-1.0507*	-1.1874	-0.8908	< 0.001
	NH White	NH White - 2 joinpoints	1	2000	2015	-0.8159*	-0.8503	-0.7777	< 0.001
	NH White	NH White - 2 joinpoints	2	2015	2019	-1.6444*	-2.0002	-1.4024	< 0.001
Kansas	NH AIAN	NH AIAN - 4 joinpoints	1	2000	2005	3.4231*	3.0977	3.9975	< 0.001
	NH AIAN	NH AIAN - 4 joinpoints	2	2005	2008	1.9477*	0.6169	3.1208	0.024
	NH AIAN	NH AIAN - 4 joinpoints	3	2008	2013	-1.5525	-1.8338	0.3487	0.052
	NH AIAN	NH AIAN - 4 joinpoints	4	2013	2019	-2.0691*	-2.7298	-1.8458	< 0.001
	NH API	NH API - 4 joinpoints	1	2000	2007	-0.6371*	-1.2503	-0.0232	0.049
	NH API	NH API - 4 joinpoints	2	2007	2012	0.3459	-1.0535	1.0331	0.26
	NH API	NH API - 4 joinpoints	3	2012	2015	-0.5104	-1.6966	0.3808	0.26
	NH API	NH API - 4 joinpoints	4	2015	2019	-2.1262*	-2.9879	-1.6426	< 0.001

	NH Black	NH Black - 1 joinpoint	1	2000	2019	-1.7545*	-1.7968	-1.7129	< 0.001
	Latino	Latino - 2 joinpoints	1	2000	2007	-1.778*	-2.2054	-1.5109	< 0.001
	Latino	Latino - 2 joinpoints	2	2007	2019	-0.5462*	-0.6776	-0.3853	< 0.001
	NH White	NH White - 2 joinpoints	1	2000	2016	-0.8323*	-0.8649	-0.7921	< 0.001
	NH White	NH White - 2 joinpoints	2	2016	2019	-1.4365*	-1.9809	-1.1136	< 0.001
Kentucky	NH AIAN	NH AIAN - 4 joinpoints	1	2000	2005	0.3615	-0.2024	0.9945	0.15
	NH AIAN	NH AIAN - 4 joinpoints	2	2005	2013	-2.2322*	-2.7348	-0.0595	0.049
	NH AIAN	NH AIAN - 4 joinpoints	3	2013	2016	-0.7231*	-2.5374	-0.2146	0.0080
	NH AIAN	NH AIAN - 4 joinpoints	4	2016	2019	-2.3633*	-3.5565	-1.4353	< 0.001
	NH API	NH API - 3 joinpoints	1	2000	2007	-0.5752*	-0.9206	-0.3796	< 0.001
	NH API	NH API - 3 joinpoints	2	2007	2015	0.138	-0.0411	0.4701	0.11
	NH API	NH API - 3 joinpoints	3	2015	2019	-1.9429*	-2.4167	-1.49	< 0.001
	NH Black	NH Black - 4 joinpoints	1	2000	2002	-1.2392*	-1.7305	-0.7837	< 0.001
	NH Black	NH Black - 4 joinpoints	2	2002	2007	-1.865*	-2.4122	-1.7475	< 0.001
	NH Black	NH Black - 4 joinpoints	3	2007	2013	-2.2582*	-2.6011	-1.569	< 0.001
	NH Black	NH Black - 4 joinpoints	4	2013	2019	-1.6983*	-2.0705	-1.3822	< 0.001
	Latino	Latino - 2 joinpoints	1	2000	2011	-2.3347*	-2.6696	-2.1065	< 0.001
	Latino	Latino - 2 joinpoints	2	2011	2019	-0.6844*	-1.0529	-0.035	0.047
	NH White	NH White - 3 joinpoints	1	2000	2006	-1.0253*	-1.2479	-0.8925	< 0.001
	NH White	NH White - 3 joinpoints	2	2006	2015	-0.5434*	-0.6234	-0.4158	< 0.001
	NH White	NH White - 3 joinpoints	3	2015	2019	-1.6076*	-1.8612	-1.3638	< 0.001

	NH AIAN	NH AIAN - 3 joinpoints	1	2000	2007	0.9621*	0.6956	1.2967	< 0.001
	NH AIAN	NH AIAN - 3 joinpoints	2	2007	2016	-0.7404*	-0.9333	-0.4618	0.0072
	NH AIAN	NH AIAN - 3 joinpoints	3	2016	2019	-2.25*	-3.4584	-1.4867	< 0.001
	NH API	NH API - 2 joinpoints	1	2000	2007	-1.4199*	-2.3026	-1.0293	< 0.001
	NH API	NH API - 2 joinpoints	2	2007	2019	-0.2274	-0.4172	0.0645	0.082
	NH Black	NH Black - 4 joinpoints	1	2000	2002	-1.1269*	-1.4801	-0.7959	< 0.001
Louisiana	NH Black	NH Black - 4 joinpoints	2	2002	2005	-1.8679*	-2.0012	-1.6191	< 0.001
	NH Black	NH Black - 4 joinpoints	3	2005	2016	-1.4937*	-1.5316	-1.3736	< 0.001
	NH Black	NH Black - 4 joinpoints	4	2016	2019	-2.0941*	-2.4399	-1.848	< 0.001
	Latino	Latino - 2 joinpoints	1	2000	2007	-3.2608*	-3.8267	-2.8526	< 0.001
	Latino	Latino - 2 joinpoints	2	2007	2019	0.3149*	0.1304	0.5318	0.0032
	NH White	NH White - 3 joinpoints	1	2000	2007	-1.4*	-1.7832	-1.218	< 0.001
	NH White	NH White - 3 joinpoints	2	2007	2014	-0.592*	-0.7906	-0.0346	0.040
	NH White	NH White - 3 joinpoints	3	2014	2019	-1.7223*	-2.1078	-1.4555	< 0.001
	NH AIAN	NH AIAN - 4 joinpoints	1	2000	2005	-0.8144*	-1.2595	-0.048	0.043
	NH AIAN	NH AIAN - 4 joinpoints	2	2005	2008	-2.7911*	-5.5726	-1.6223	< 0.001
Maine	NH AIAN	NH AIAN - 4 joinpoints	3	2008	2011	-6.0835*	-6.6353	-1.2417	0.0016
	NH AIAN	NH AIAN - 4 joinpoints	4	2011	2019	-1.1559*	-1.4403	-0.767	0.0036
	NH API	NH API - 3 joinpoints	1	2000	2006	-2.491*	-3.0187	-2.1741	< 0.001
	NH API	NH API - 3 joinpoints	2	2006	2015	-1.1935*	-1.3584	-0.5683	0.0020
	NH API	NH API - 3 joinpoints	3	2015	2019	-2.1304*	-3.0736	-1.6398	< 0.001

	NH Black	NH Black - 4 joinpoints	1	2000	2006	-3.7588*	-3.9822	-3.5673	< 0.001
	NH Black	NH Black - 4 joinpoints	2	2006	2009	-0.6749*	-1.1797	-0.3406	< 0.001
	NH Black	NH Black - 4 joinpoints	3	2009	2014	-1.9173*	-2.4151	-1.7096	< 0.001
	NH Black	NH Black - 4 joinpoints	4	2014	2019	-1.2204*	-1.4701	-0.5435	< 0.001
	Latino	Latino - 3 joinpoints	1	2000	2007	-1.3547*	-2.0887	-1.0881	0.0044
	Latino	Latino - 3 joinpoints	2	2007	2012	-0.3002	-0.7016	0.4746	0.45
	Latino	Latino - 3 joinpoints	3	2012	2019	-1.0555*	-1.8109	-0.8506	< 0.001
	NH White	NH White - 2 joinpoints	1	2000	2016	-1.3002*	-1.3394	-1.2322	< 0.001
	NH White	NH White - 2 joinpoints	2	2016	2019	-1.8976*	-2.7195	-1.4782	< 0.001
Maryland	NH AIAN	NH AIAN - 3 joinpoints	1	2000	2005	-2.3076*	-3.0109	-0.7264	0.034
	NH AIAN	NH AIAN - 3 joinpoints	2	2005	2009	-3.6697*	-4.7415	-0.5952	0.0044
	NH AIAN	NH AIAN - 3 joinpoints	3	2009	2019	-1.7586*	-3.727	-0.8392	0.0072
	NH API	NH API - 2 joinpoints	1	2000	2015	-1.2162*	-1.2907	-0.9497	0.021
	NH API	NH API - 2 joinpoints	2	2015	2019	-1.8524*	-3.2092	-1.3645	< 0.001
	NH Black	NH Black - 2 joinpoints	1	2000	2008	-2.5317*	-3.0443	-2.3614	< 0.001
	NH Black	NH Black - 2 joinpoints	2	2008	2019	-2.0856*	-2.1891	-1.8176	< 0.001
	Latino	Latino - 3 joinpoints	1	2000	2002	1.6662*	0.3098	3.0163	0.0076
	Latino	Latino - 3 joinpoints	2	2002	2015	0.1015	-0.1514	0.2297	0.23
	Latino	Latino - 3 joinpoints	3	2015	2019	-1.3403*	-2.5743	-0.7289	0.0068
	NH White	NH White - 3 joinpoints	1	2000	2013	-1.7462*	-1.8377	-1.7005	< 0.001
	NH White	NH White - 3 joinpoints	2	2013	2016	-1.2055*	-1.5916	-1.0287	< 0.001

	NH White	NH White - 3 joinpoints	3	2016	2019	-2.0982*	-2.6655	-1.8032	< 0.001
Massachusetts	NH AIAN	NH AIAN - 3 joinpoints	1	2000	2006	-0.3001	-0.6221	0.0773	0.095
	NH AIAN	NH AIAN - 3 joinpoints	2	2006	2016	-2.2178*	-2.3743	-2.0212	< 0.001
	NH AIAN	NH AIAN - 3 joinpoints	3	2016	2019	-3.7695*	-4.9618	-3.0728	< 0.001
	NH API	NH API - 2 joinpoints	1	2000	2015	-0.9447*	-1.0443	-0.8421	< 0.001
	NH API	NH API - 2 joinpoints	2	2015	2019	-3.3375*	-4.2595	-2.6774	< 0.001
	NH Black	NH Black - 4 joinpoints	1	2000	2002	-2.3438*	-2.8563	-1.8268	< 0.001
	NH Black	NH Black - 4 joinpoints	2	2002	2006	-3.5395*	-3.8527	-3.3291	< 0.001
	NH Black	NH Black - 4 joinpoints	3	2006	2012	-3.0068*	-3.2069	-2.6904	< 0.001
	NH Black	NH Black - 4 joinpoints	4	2012	2019	-2.524*	-2.6454	-2.1834	< 0.001
	Latino	Latino - 3 joinpoints	1	2000	2007	-1.975*	-2.5053	-1.6175	0.0012
	Latino	Latino - 3 joinpoints	2	2007	2017	-0.3047	-1.3048	0.4367	0.13
	Latino	Latino - 3 joinpoints	3	2017	2019	-1.8936*	-3.0902	-0.4993	< 0.001
	NH White	NH White - 3 joinpoints	1	2000	2009	-1.9186*	-2.0404	-1.837	< 0.001
	NH White	NH White - 3 joinpoints	2	2009	2015	-1.3299*	-1.4704	-0.9611	< 0.001
	NH White	NH White - 3 joinpoints	3	2015	2019	-1.8602*	-2.2837	-1.654	< 0.001
Michigan	NH AIAN	NH AIAN - 2 joinpoints	1	2000	2006	-0.5878	-1.1569	0.3807	0.12
	NH AIAN	NH AIAN - 2 joinpoints	2	2006	2019	-2.2852*	-2.544	-2.0962	< 0.001
	NH API	NH API - 4 joinpoints	1	2000	2007	-1.3309*	-2.1633	-0.6151	0.026
	NH API	NH API - 4 joinpoints	2	2007	2011	-0.4164	-1.5137	0.2012	0.14
	NH API	NH API - 4 joinpoints	3	2011	2015	-1.5203*	-2.3341	-0.5016	0.011
	NH API	NH API - 4 joinpoints	4	2015	2019	-2.792*	-3.8344	-2.218	< 0.001

	NH Black	NH Black - 2 joinpoints	1	2000	2013	-1.4642*	-1.5128	-1.4091	< 0.001
	NH Black	NH Black - 2 joinpoints	2	2013	2019	-2.1437*	-2.3361	-1.9896	< 0.001
	Latino	Latino - 3 joinpoints	1	2000	2005	0.3432	-0.0528	0.964	0.082
	Latino	Latino - 3 joinpoints	2	2005	2013	-1.0792*	-1.3305	-0.8057	< 0.001
	Latino	Latino - 3 joinpoints	3	2013	2019	-2.0599*	-2.6049	-1.7806	< 0.001
	NH White	NH White - 2 joinpoints	1	2000	2016	-1.103*	-1.1357	-1.0664	< 0.001
	NH White	NH White - 2 joinpoints	2	2016	2019	-1.9627*	-2.539	-1.6136	< 0.001
Minnesota	NH AIAN	NH AIAN - 2 joinpoints	1	2000	2007	0.0885	-0.3441	0.7545	0.67
	NH AIAN	NH AIAN - 2 joinpoints	2	2007	2019	-1.3906*	-1.6586	-1.1909	< 0.001
	NH API	NH API - 3 joinpoints	1	2000	2007	-1.4926*	-1.7478	-1.3134	< 0.001
	NH API	NH API - 3 joinpoints	2	2007	2015	-0.3948*	-0.5593	-0.1678	0.0064
	NH API	NH API - 3 joinpoints	3	2015	2019	-2.5345*	-2.9013	-2.0908	< 0.001
	NH Black	NH Black - 4 joinpoints	1	2000	2002	-2.2514*	-2.9344	-1.6378	< 0.001
	NH Black	NH Black - 4 joinpoints	2	2002	2006	-3.1466*	-3.6817	-1.2544	< 0.001
	NH Black	NH Black - 4 joinpoints	3	2006	2012	-1.2551*	-2.3096	-0.8591	< 0.001
	NH Black	NH Black - 4 joinpoints	4	2012	2019	-2.0514*	-2.2609	-1.7315	< 0.001
	Latino	Latino - 2 joinpoints	1	2000	2007	-2.1743*	-2.6305	-1.9062	< 0.001
	Latino	Latino - 2 joinpoints	2	2007	2019	-1.1465*	-1.2757	-0.9784	< 0.001
	NH White	NH White - 3 joinpoints	1	2000	2007	-1.3471*	-1.666	-1.2494	< 0.001
	NH White	NH White - 3 joinpoints	2	2007	2015	-1.0933*	-1.178	-0.7929	< 0.001
	NH White	NH White - 3 joinpoints	3	2015	2019	-1.8167*	-2.1577	-1.5891	< 0.001

Mississippi	NH AIAN	NH AIAN - 2 joinpoints	1	2000	2008	-0.9248	-1.2481	0.137	0.060
	NH AIAN	NH AIAN - 2 joinpoints	2	2008	2019	-1.6302*	-2.5124	-1.4268	< 0.001
	NH API	NH API - 1 joinpoint	1	2000	2019	-0.514*	-0.6023	-0.4245	< 0.001
	NH Black	NH Black - 3 joinpoints	1	2000	2007	-0.8729*	-0.9879	-0.6732	< 0.001
	NH Black	NH Black - 3 joinpoints	2	2007	2014	-1.4429*	-1.5935	-1.1977	< 0.001
	NH Black	NH Black - 3 joinpoints	3	2014	2019	-1.9131*	-2.2971	-1.7312	< 0.001
	Latino	Latino - 2 joinpoints	1	2000	2008	-1.8296*	-2.3412	-1.4514	< 0.001
	Latino	Latino - 2 joinpoints	2	2008	2019	0.4263*	0.1951	0.7323	< 0.001
	NH White	NH White - 3 joinpoints	1	2000	2007	-0.7453*	-0.9789	-0.6101	< 0.001
	NH White	NH White - 3 joinpoints	2	2007	2014	-0.0861	-0.2414	0.1927	0.36
	NH White	NH White - 3 joinpoints	3	2014	2019	-1.4677*	-1.6939	-1.2337	< 0.001
Missouri	NH AIAN	NH AIAN - 3 joinpoints	1	2000	2006	1.1585*	0.6552	1.9025	0.012
	NH AIAN	NH AIAN - 3 joinpoints	2	2006	2016	-1.2172*	-1.4412	-0.7463	0.024
	NH AIAN	NH AIAN - 3 joinpoints	3	2016	2019	-3.0754*	-5.3406	-1.9192	< 0.001
	NH API	NH API - 4 joinpoints	1	2000	2007	-0.8573*	-1.2643	-0.6321	0.011
	NH API	NH API - 4 joinpoints	2	2007	2011	0.4577	-0.5124	1.0364	0.10
	NH API	NH API - 4 joinpoints	3	2011	2015	-0.5779	-1.1993	0.058	0.068
	NH API	NH API - 4 joinpoints	4	2015	2019	-2.0314*	-2.7433	-1.6468	< 0.001
	NH Black	NH Black - 2 joinpoints	1	2000	2012	-1.7068*	-1.7796	-1.6512	< 0.001
	NH Black	NH Black - 2 joinpoints	2	2012	2019	-1.2824*	-1.4031	-1.0839	< 0.001
	Latino	Latino - 3 joinpoints	1	2000	2007	-3.1832*	-4.1094	-2.1571	< 0.001
	Latino	Latino - 3 joinpoints	2	2007	2011	-1.6725*	-3.8925	-0.778	0.0020

	Latino	Latino - 3 joinpoints	3	2011	2019	-1.1285*	-2.2172	-0.25	0.020
	NH White	NH White - 2 joinpoints	1	2000	2016	-0.9451*	-0.9749	-0.9118	< 0.001
	NH White	NH White - 2 joinpoints	2	2016	2019	-1.6596*	-2.2341	-1.3484	< 0.001
Montana	NH AIAN	NH AIAN - 4 joinpoints	1	2000	2007	-0.5369*	-0.7587	-0.2489	0.0056
	NH AIAN	NH AIAN - 4 joinpoints	2	2007	2011	-2.2413*	-2.8807	-1.71	0.0052
	NH AIAN	NH AIAN - 4 joinpoints	3	2011	2016	-0.7826	-1.0954	0.0451	0.064
	NH AIAN	NH AIAN - 4 joinpoints	4	2016	2019	-2.3537*	-3.4207	-1.7468	< 0.001
	NH API	NH API - 2 joinpoints	1	2000	2015	-0.7538*	-0.8397	-0.6532	< 0.001
	NH API	NH API - 2 joinpoints	2	2015	2019	-1.9798*	-2.9109	-1.4575	< 0.001
	NH Black	NH Black - 1 joinpoint	1	2000	2019	-2.506*	-2.682	-2.3328	< 0.001
	Latino	Latino - 2 joinpoints	1	2000	2007	-2.3074*	-2.8948	-2.011	< 0.001
	Latino	Latino - 2 joinpoints	2	2007	2019	-1.4345*	-1.5793	-1.2245	< 0.001
	NH White	NH White - 2 joinpoints	1	2000	2015	-1.5507*	-1.5913	-1.5043	< 0.001
	NH White	NH White - 2 joinpoints	2	2015	2019	-2.1868*	-2.637	-1.9367	< 0.001
Nebraska	NH AIAN	NH AIAN - 2 joinpoints	1	2000	2005	0.0533	-0.5766	1.1874	0.86
	NH AIAN	NH AIAN - 2 joinpoints	2	2005	2019	-1.5016*	-1.7078	-1.35	< 0.001
	NH API	NH API - 4 joinpoints	1	2000	2007	-1.4011*	-1.8866	-1.0556	0.0012
	NH API	NH API - 4 joinpoints	2	2007	2012	1.019	-1.5655	1.7471	0.19
	NH API	NH API - 4 joinpoints	3	2012	2015	-0.2014	-1.1254	1.0346	0.71
	NH API	NH API - 4 joinpoints	4	2015	2019	-1.8147*	-2.6187	-1.3499	< 0.001
	NH Black	NH Black - 4 joinpoints	1	2000	2002	-1.1742*	-1.7877	-0.726	< 0.001
	NH Black	NH Black - 4 joinpoints	2	2002	2006	-2.0325*	-2.3876	-0.9989	< 0.001

	NH Black	NH Black - 4 joinpoints	3	2006	2012	-1.0463*	-2.3766	-0.8255	< 0.001
	NH Black	NH Black - 4 joinpoints	4	2012	2019	-2.4089*	-2.5408	-2.2493	< 0.001
	Latino	Latino - 2 joinpoints	1	2000	2007	-2.4741*	-2.9494	-2.1867	< 0.001
	Latino	Latino - 2 joinpoints	2	2007	2019	-1.4521*	-1.5934	-1.2712	< 0.001
	NH White	NH White - 2 joinpoints	1	2000	2016	-1.098*	-1.1295	-1.065	< 0.001
	NH White	NH White - 2 joinpoints	2	2016	2019	-2.021*	-2.5142	-1.6761	< 0.001
Nevada	NH AIAN	NH AIAN - 3 joinpoints	1	2000	2005	0.6847*	0.2454	1.5328	0.0020
	NH AIAN	NH AIAN - 3 joinpoints	2	2005	2015	-0.5611*	-0.8134	-0.3597	< 0.001
	NH AIAN	NH AIAN - 3 joinpoints	3	2015	2019	-2.3514*	-3.4684	-1.7531	< 0.001
	NH API	NH API - 4 joinpoints	1	2000	2009	-1.5629*	-2.1856	-0.9052	< 0.001
	NH API	NH API - 4 joinpoints	2	2009	2013	-0.8429*	-2.0362	-0.5163	0.0016
	NH API	NH API - 4 joinpoints	3	2013	2016	0.2822	-0.4013	0.7917	0.50
	NH API	NH API - 4 joinpoints	4	2016	2019	-1.599*	-2.64	-1.0503	< 0.001
	NH Black	NH Black - 3 joinpoints	1	2000	2002	-1.1315*	-1.7832	-0.579	< 0.001
	NH Black	NH Black - 3 joinpoints	2	2002	2012	-1.8436*	-2.2025	-1.1826	< 0.001
	NH Black	NH Black - 3 joinpoints	3	2012	2019	-1.069*	-1.2761	-0.7676	< 0.001
	Latino	Latino - 2 joinpoints	1	2000	2002	-0.1485	-1.0609	0.7005	0.44
	Latino	Latino - 2 joinpoints	2	2002	2019	-1.0724*	-1.8358	-0.9354	< 0.001
	NH White	NH White - 4 joinpoints	1	2000	2006	-1.1189*	-1.2753	-0.9592	< 0.001
	NH White	NH White - 4 joinpoints	2	2006	2013	-2.1382*	-2.39	-2.0034	< 0.001
	NH White	NH White - 4 joinpoints	3	2013	2016	-1.4319*	-1.8095	-1.2358	< 0.001

	NH White	NH White - 4 joinpoints	4	2016	2019	-2.2859*	-2.8033	-2.0048	< 0.001
New Hampshire	NH AIAN	NH AIAN - 2 joinpoints	1	2000	2005	0.5317	-0.3021	1.6984	0.22
	NH AIAN	NH AIAN - 2 joinpoints	2	2005	2019	-2.2442*	-2.4696	-2.0567	< 0.001
	NH API	NH API - 4 joinpoints	1	2000	2007	-1.193*	-1.6549	-0.6995	0.0028
	NH API	NH API - 4 joinpoints	2	2007	2012	-0.0651	-1.5616	0.5692	0.79
	NH API	NH API - 4 joinpoints	3	2012	2015	-1.5509	-2.185	0.2473	0.10
	NH API	NH API - 4 joinpoints	4	2015	2019	-2.5839*	-3.3649	-2.122	< 0.001
	NH Black	NH Black - 3 joinpoints	1	2000	2007	-3.7715*	-4.0737	-3.5704	< 0.001
	NH Black	NH Black - 3 joinpoints	2	2007	2012	-1.634*	-3.1025	-1.2718	< 0.001
	NH Black	NH Black - 3 joinpoints	3	2012	2019	-0.9092*	-1.1102	-0.3803	0.010
	Latino	Latino - 2 joinpoints	1	2000	2007	-2.1078*	-2.5685	-1.7579	< 0.001
	Latino	Latino - 2 joinpoints	2	2007	2019	0.0817	-0.0826	0.266	0.30
	NH White	NH White - 2 joinpoints	1	2000	2002	-0.722*	-1.3574	-0.0747	0.036
	NH White	NH White - 2 joinpoints	2	2002	2019	-1.3892*	-2.0399	-1.3454	< 0.001
New Jersey	NH AIAN	NH AIAN - 4 joinpoints	1	2000	2006	-1.108*	-1.4582	-0.7542	0.0016
	NH AIAN	NH AIAN - 4 joinpoints	2	2006	2012	-3.9086*	-4.6026	-3.5852	< 0.001
	NH AIAN	NH AIAN - 4 joinpoints	3	2012	2015	-2.1974*	-2.9782	-1.7147	< 0.001
	NH AIAN	NH AIAN - 4 joinpoints	4	2015	2019	-4.0776*	-5.0496	-3.6549	< 0.001
	NH API	NH API - 2 joinpoints	1	2000	2015	-1.0939*	-1.1663	-1.017	< 0.001
	NH API	NH API - 2 joinpoints	2	2015	2019	-2.5407*	-3.2866	-2.0933	< 0.001
	NH Black	NH Black - 4 joinpoints	1	2000	2002	-1.9388*	-2.4516	-1.4973	< 0.001

	NH Black	NH Black - 4 joinpoints	2	2002	2006	-2.6734*	-2.994	-1.8874	< 0.001
	NH Black	NH Black - 4 joinpoints	3	2006	2016	-1.9033*	-1.9721	-1.7773	< 0.001
	NH Black	NH Black - 4 joinpoints	4	2016	2019	-2.7918*	-3.1739	-2.4792	< 0.001
	Latino	Latino - 3 joinpoints	1	2000	2007	-1.7246*	-2.6212	-1.462	< 0.001
	Latino	Latino - 3 joinpoints	2	2007	2014	-0.8934*	-1.18	-0.0045	0.050
	Latino	Latino - 3 joinpoints	3	2014	2019	-2.5469*	-3.233	-2.1125	< 0.001
	NH White	NH White - 2 joinpoints	1	2000	2016	-1.7536*	-1.7937	-1.5708	< 0.001
	NH White	NH White - 2 joinpoints	2	2016	2019	-2.2607*	-3.1079	-1.8364	< 0.001
New Mexico	NH AIAN	NH AIAN - 4 joinpoints	1	2000	2008	-0.7579*	-0.8756	-0.4896	0.010
	NH AIAN	NH AIAN - 4 joinpoints	2	2008	2012	-1.4393*	-1.8756	-1.1125	< 0.001
	NH AIAN	NH AIAN - 4 joinpoints	3	2012	2016	-0.329	-0.6722	0.1721	0.20
	NH AIAN	NH AIAN - 4 joinpoints	4	2016	2019	-2.0485*	-2.7779	-1.5598	< 0.001
	NH API	NH API - 3 joinpoints	1	2000	2004	-2.1088*	-3.3602	-1.5191	< 0.001
	NH API	NH API - 3 joinpoints	2	2004	2015	-0.6188*	-0.7544	-0.3362	0.0084
	NH API	NH API - 3 joinpoints	3	2015	2019	-2.1448*	-3.0368	-1.6008	< 0.001
	NH Black	NH Black - 4 joinpoints	1	2000	2002	-1.3501*	-1.9357	-0.9032	< 0.001
	NH Black	NH Black - 4 joinpoints	2	2002	2005	-2.2369*	-2.4464	-1.4073	< 0.001
	NH Black	NH Black - 4 joinpoints	3	2005	2013	-1.4979*	-2.0873	-1.1924	< 0.001
	NH Black	NH Black - 4 joinpoints	4	2013	2019	-1.9368*	-2.1659	-1.6933	< 0.001
	Latino	Latino - 2 joinpoints	1	2000	2005	-0.8323*	-1.1416	-0.174	0.032
	Latino	Latino - 2 joinpoints	2	2005	2019	-1.4884*	-1.5976	-1.412	< 0.001

	NH White	NH White - 3 joinpoints	1	2000	2007	-1.2985*	-1.3793	-0.9879	< 0.001
	NH White	NH White - 3 joinpoints	2	2007	2015	-1.4975*	-1.6835	-1.4239	< 0.001
	NH White	NH White - 3 joinpoints	3	2015	2019	-2.2009*	-2.5211	-2.0138	< 0.001
New York	NH AIAN	NH AIAN - 4 joinpoints	1	2000	2007	-0.1357	-0.4405	0.1379	0.29
	NH AIAN	NH AIAN - 4 joinpoints	2	2007	2012	-3.389*	-4.0641	-2.8773	0.013
	NH AIAN	NH AIAN - 4 joinpoints	3	2012	2015	-0.7577*	-1.6079	-0.2409	0.0048
	NH AIAN	NH AIAN - 4 joinpoints	4	2015	2019	-2.6502*	-3.606	-2.2289	< 0.001
	NH API	NH API - 4 joinpoints	1	2000	2006	-1.5959*	-2.258	-1.287	< 0.001
	NH API	NH API - 4 joinpoints	2	2006	2011	-0.1534	-1.0912	0.6099	0.38
	NH API	NH API - 4 joinpoints	3	2011	2015	-0.8708*	-1.7101	-0.3819	0.0028
	NH API	NH API - 4 joinpoints	4	2015	2019	-2.9762*	-3.6343	-2.5857	< 0.001
	NH Black	NH Black - 3 joinpoints	1	2000	2006	-2.5221*	-2.8936	-2.2984	< 0.001
	NH Black	NH Black - 3 joinpoints	2	2006	2013	-1.3518*	-1.5488	-1.0083	< 0.001
	NH Black	NH Black - 3 joinpoints	3	2013	2019	-2.9275*	-3.1556	-2.689	< 0.001
	Latino	Latino - 3 joinpoints	1	2000	2007	-1.6771*	-2.4407	-1.4015	< 0.001
	Latino	Latino - 3 joinpoints	2	2007	2013	-0.5738	-0.9754	0.3127	0.14
	Latino	Latino - 3 joinpoints	3	2013	2019	-2.9315*	-3.35	-2.5672	< 0.001
	NH White	NH White - 2 joinpoints	1	2000	2015	-1.5761*	-1.6184	-1.5298	< 0.001
	NH White	NH White - 2 joinpoints	2	2015	2019	-2.5443*	-2.9918	-2.2619	< 0.001
North Carolina	NH AIAN	NH AIAN - 4 joinpoints	1	2000	2008	-0.3698*	-0.5636	-0.1531	0.016
	NH AIAN	NH AIAN - 4 joinpoints	2	2008	2012	-1.7005*	-2.3412	-0.9575	0.028

	NH AIAN	NH AIAN - 4 joinpoints	3	2012	2015	-0.3477	-1.0341	0.1064	0.098
	NH AIAN	NH AIAN - 4 joinpoints	4	2015	2019	-1.7705*	-2.5517	-1.4538	< 0.001
	NH API	NH API - 2 joinpoints	1	2000	2015	-0.6156*	-0.6886	-0.53	< 0.001
	NH API	NH API - 2 joinpoints	2	2015	2019	-1.7701*	-2.5626	-1.3147	< 0.001
	NH Black	NH Black - 2 joinpoints	1	2000	2002	-1.1374*	-1.6112	-0.7868	< 0.001
	NH Black	NH Black - 2 joinpoints	2	2002	2019	-1.7288*	-1.7729	-1.7063	< 0.001
	Latino	Latino - 2 joinpoints	1	2000	2009	0.3389	-0.1765	0.6088	0.11
	Latino	Latino - 2 joinpoints	2	2009	2019	1.459*	1.2195	1.9001	< 0.001
	NH White	NH White - 3 joinpoints	1	2000	2006	-0.8165*	-0.939	-0.5178	0.0020
	NH White	NH White - 3 joinpoints	2	2006	2016	-1.1322*	-1.2353	-1.0624	< 0.001
	NH White	NH White - 3 joinpoints	3	2016	2019	-2.1331*	-2.6811	-1.7848	< 0.001
	NH AIAN	NH AIAN - 4 joinpoints	1	2000	2007	0.0566	-0.1712	0.2754	0.49
	NH AIAN	NH AIAN - 4 joinpoints	2	2007	2011	-2.4896*	-3.0376	-1.9121	0.016
	NH AIAN	NH AIAN - 4 joinpoints	3	2011	2016	-1.2022*	-1.4984	-0.5075	< 0.001
	NH AIAN	NH AIAN - 4 joinpoints	4	2016	2019	-2.4363*	-3.3473	-1.8853	< 0.001
North Dakota	NH API	NH API - 3 joinpoints	1	2000	2006	-1.1609*	-1.7011	-0.801	< 0.001
	NH API	NH API - 3 joinpoints	2	2006	2015	0.217*	0.0019	0.5622	0.048
	NH API	NH API - 3 joinpoints	3	2015	2019	-1.9625*	-2.8417	-1.3602	< 0.001
	NH Black	NH Black - 3 joinpoints	1	2000	2003	-1.9688*	-2.797	-0.7451	0.0040
	NH Black	NH Black - 3 joinpoints	2	2003	2006	-3.3278*	-3.8189	-0.6166	< 0.001
	NH Black	NH Black - 3 joinpoints	3	2006	2019	-1.2745*	-2.2625	-0.8501	< 0.001

	Latino	Latino - 1 joinpoint	1	2000	2019	-0.9333*	-1.0598	-0.8088	< 0.001
	NH White	NH White - 3 joinpoints	1	2000	2006	-1.3704*	-1.8819	-1.209	< 0.001
	NH White	NH White - 3 joinpoints	2	2006	2014	-0.9811*	-1.0989	-0.6257	< 0.001
	NH White	NH White - 3 joinpoints	3	2014	2019	-2.0532*	-2.3279	-1.8229	< 0.001
Ohio	NH AIAN	NH AIAN - 2 joinpoints	1	2000	2005	0.4156	-0.2479	1.4715	0.21
	NH AIAN	NH AIAN - 2 joinpoints	2	2005	2019	-1.5909*	-1.7822	-1.4378	< 0.001
	NH API	NH API - 3 joinpoints	1	2000	2006	-0.6532*	-1.5657	-0.3135	< 0.001
	NH API	NH API - 3 joinpoints	2	2006	2015	0.2788*	0.0903	0.7816	0.0072
	NH API	NH API - 3 joinpoints	3	2015	2019	-2.0306*	-2.6372	-1.4795	< 0.001
	NH Black	NH Black - 2 joinpoints	1	2000	2002	-0.6541*	-1.3803	-0.2369	< 0.001
	NH Black	NH Black - 2 joinpoints	2	2002	2019	-1.8617*	-1.9081	-1.828	< 0.001
	Latino	Latino - 3 joinpoints	1	2000	2007	-2.2501*	-2.5926	-1.991	< 0.001
	Latino	Latino - 3 joinpoints	2	2007	2016	-0.7399*	-0.9147	-0.1722	0.028
	Latino	Latino - 3 joinpoints	3	2016	2019	-1.8079*	-2.9624	-1.0951	< 0.001
	NH White	NH White - 3 joinpoints	1	2000	2007	-1.0708*	-1.3836	-0.985	< 0.001
	NH White	NH White - 3 joinpoints	2	2007	2016	-0.8896*	-0.9493	-0.6539	< 0.001
	NH White	NH White - 3 joinpoints	3	2016	2019	-1.6235*	-2.1021	-1.3159	< 0.001
Oklahoma	NH AIAN	NH AIAN - 4 joinpoints	1	2000	2005	3.3332*	3.0078	3.8405	< 0.001
	NH AIAN	NH AIAN - 4 joinpoints	2	2005	2008	1.4488*	0.2862	2.5808	0.021
	NH AIAN	NH AIAN - 4 joinpoints	3	2008	2015	-0.3155*	-0.6076	-0.0716	0.024
	NH AIAN	NH AIAN - 4 joinpoints	4	2015	2019	-1.6751*	-2.4548	-1.2573	< 0.001

	NH API	NH API - 4 joinpoints	1	2000	2007	-0.7828*	-1.2897	-0.2546	0.037
	NH API	NH API - 4 joinpoints	2	2007	2012	0.3146	-1.2013	0.9428	0.32
	NH API	NH API - 4 joinpoints	3	2012	2015	-0.5098	-1.3606	0.4475	0.32
	NH API	NH API - 4 joinpoints	4	2015	2019	-1.6617*	-2.4162	-1.2128	< 0.001
	NH Black	NH Black - 3 joinpoints	1	2000	2002	-0.3848*	-0.9274	-0.0662	0.016
	NH Black	NH Black - 3 joinpoints	2	2002	2011	-1.4283*	-1.5331	-1.3509	< 0.001
	NH Black	NH Black - 3 joinpoints	3	2011	2019	-2.1154*	-2.2225	-2.0346	< 0.001
	Latino	Latino - 3 joinpoints	1	2000	2007	-1.9069*	-2.4932	-1.5989	< 0.001
	Latino	Latino - 3 joinpoints	2	2007	2012	-0.369	-1.3686	0.4724	0.31
	Latino	Latino - 3 joinpoints	3	2012	2019	-1.0583*	-1.843	-0.7993	< 0.001
	NH White	NH White - 2 joinpoints	1	2000	2015	-0.7674*	-0.804	-0.7293	< 0.001
	NH White	NH White - 2 joinpoints	2	2015	2019	-1.6226*	-1.992	-1.3774	< 0.001
Oregon	NH AIAN	NH AIAN - 3 joinpoints	1	2000	2002	0.898	-0.5241	1.9332	0.23
	NH AIAN	NH AIAN - 3 joinpoints	2	2002	2016	-1.6127*	-1.7202	-1.4971	< 0.001
	NH AIAN	NH AIAN - 3 joinpoints	3	2016	2019	-2.9341*	-4.3845	-2.1761	< 0.001
	NH API	NH API - 3 joinpoints	1	2000	2007	-1.7143*	-2.2923	-1.4997	< 0.001
	NH API	NH API - 3 joinpoints	2	2007	2015	-1.0056*	-1.1797	-0.3168	0.0020
	NH API	NH API - 3 joinpoints	3	2015	2019	-1.9399*	-2.7606	-1.5313	< 0.001
	NH Black	NH Black - 3 joinpoints	1	2000	2006	-2.0478*	-2.4027	-1.8043	< 0.001
	NH Black	NH Black - 3 joinpoints	2	2006	2012	-0.5391*	-0.8283	-0.0552	0.040
	NH Black	NH Black - 3 joinpoints	3	2012	2019	-2.465*	-2.6677	-2.255	< 0.001
	Latino	Latino - 3 joinpoints	1	2000	2007	-1.0047*	-1.624	-0.7522	< 0.001
	Latino	Latino - 3 joinpoints	2	2007	2013	0.0657	-0.2855	0.8815	0.64

	Latino	Latino - 3 joinpoints	3	2013	2019	-1.7631*	-2.1643	-1.4375	< 0.001
	NH White	NH White - 3 joinpoints	1	2000	2002	-0.6468*	-1.2147	-0.2213	< 0.001
	NH White	NH White - 3 joinpoints	2	2002	2014	-1.2924*	-1.6325	-1.2487	< 0.001
	NH White	NH White - 3 joinpoints	3	2014	2019	-2.3503*	-2.6034	-2.1314	< 0.001
Pennsylvania	NH AIAN	NH AIAN - 2 joinpoints	1	2000	2005	-0.8622	-1.5515	0.4531	0.12
	NH AIAN	NH AIAN - 2 joinpoints	2	2005	2019	-2.4007*	-2.6291	-2.2377	< 0.001
	NH API	NH API - 4 joinpoints	1	2000	2006	-1.3904*	-1.945	-1.1276	< 0.001
	NH API	NH API - 4 joinpoints	2	2006	2011	-0.0237	-0.4534	0.6867	0.95
	NH API	NH API - 4 joinpoints	3	2011	2015	-1.133*	-1.6952	-0.5063	0.0084
	NH API	NH API - 4 joinpoints	4	2015	2019	-2.5948*	-3.3209	-2.2086	< 0.001
	NH Black	NH Black - 2 joinpoints	1	2000	2014	-1.8421*	-1.8919	-1.7833	< 0.001
	NH Black	NH Black - 2 joinpoints	2	2014	2019	-2.5891*	-2.9154	-2.3707	< 0.001
	Latino	Latino - 3 joinpoints	1	2000	2007	-3.0336*	-3.2911	-2.7977	< 0.001
	Latino	Latino - 3 joinpoints	2	2007	2013	0.319	-0.002	0.7843	0.051
	Latino	Latino - 3 joinpoints	3	2013	2019	-1.7428*	-2.1268	-1.4117	< 0.001
	NH White	NH White - 3 joinpoints	1	2000	2002	-0.8123*	-1.2431	-0.4232	< 0.001
	NH White	NH White - 3 joinpoints	2	2002	2016	-1.3182*	-1.3774	-1.2779	< 0.001
	NH White	NH White - 3 joinpoints	3	2016	2019	-1.8834*	-2.4538	-1.5972	< 0.001
Rhode Island	NH AIAN	NH AIAN - 3 joinpoints	1	2000	2007	-0.2651	-0.5539	0.0156	0.055
	NH AIAN	NH AIAN - 3 joinpoints	2	2007	2016	-2.591*	-2.745	-2.0749	0.012
	NH AIAN	NH AIAN - 3 joinpoints	3	2016	2019	-3.7229*	-4.8414	-2.987	< 0.001
	NH API	NH API - 3 joinpoints	1	2000	2012	-0.5013	-0.9746	0.3089	0.068

	NH API	NH API - 3 joinpoints	2	2012	2015	-1.9875*	-2.9157	-0.04	0.037
	NH API	NH API - 3 joinpoints	3	2015	2019	-2.842*	-3.9507	-2.2032	< 0.001
	NH Black	NH Black - 3 joinpoints	1	2000	2003	-3.8872*	-4.2349	-3.3644	< 0.001
	NH Black	NH Black - 3 joinpoints	2	2003	2013	-4.4285*	-4.5907	-4.3656	< 0.001
	NH Black	NH Black - 3 joinpoints	3	2013	2019	-3.048*	-3.1985	-2.8489	< 0.001
	Latino	Latino - 3 joinpoints	1	2000	2002	-0.4116	-1.6761	0.541	0.30
	Latino	Latino - 3 joinpoints	2	2002	2007	-2.0477*	-2.7969	-0.6661	< 0.001
	Latino	Latino - 3 joinpoints	3	2007	2019	-0.827*	-1.0041	-0.6477	0.0036
	NH White	NH White - 3 joinpoints	1	2000	2009	-1.4177*	-1.6121	-1.3359	< 0.001
	NH White	NH White - 3 joinpoints	2	2009	2016	-1.108*	-1.218	-0.7709	< 0.001
	NH White	NH White - 3 joinpoints	3	2016	2019	-1.6442*	-2.1978	-1.3492	< 0.001
South Carolina	NH AIAN	NH AIAN - 4 joinpoints	1	2000	2008	-0.6588*	-0.8107	-0.4331	0.0028
	NH AIAN	NH AIAN - 4 joinpoints	2	2008	2012	-1.8038*	-2.4041	-1.3889	0.0016
	NH AIAN	NH AIAN - 4 joinpoints	3	2012	2016	-0.5062	-0.9062	0.1455	0.12
	NH AIAN	NH AIAN - 4 joinpoints	4	2016	2019	-2.2232*	-3.1001	-1.6986	< 0.001
	NH API	NH API - 3 joinpoints	1	2000	2010	-0.2202	-0.5815	0.6033	0.15
	NH API	NH API - 3 joinpoints	2	2010	2015	-0.7677	-1.5849	0.0385	0.056
	NH API	NH API - 3 joinpoints	3	2015	2019	-1.8509*	-2.9796	-1.1782	< 0.001
	NH Black	NH Black - 2 joinpoints	1	2000	2002	-0.8664*	-1.3535	-0.507	< 0.001
	NH Black	NH Black - 2 joinpoints	2	2002	2019	-1.5093*	-1.5497	-1.4858	< 0.001
	Latino	Latino - 2 joinpoints	1	2000	2010	-0.1477	-0.369	0.0121	0.067
	Latino	Latino - 2 joinpoints	2	2010	2019	0.7627*	0.5798	1.0403	< 0.001

	NH White	NH White - 2 joinpoints	1	2000	2015	-0.7897*	-0.8369	-0.7378	< 0.001
	NH White	NH White - 2 joinpoints	2	2015	2019	-1.8345*	-2.3166	-1.5265	< 0.001
South Dakota	NH AIAN	NH AIAN - 4 joinpoints	1	2000	2002	0.095	-0.9863	1.0346	0.94
	NH AIAN	NH AIAN - 4 joinpoints	2	2002	2013	-1.4539*	-1.9998	-1.3743	< 0.001
	NH AIAN	NH AIAN - 4 joinpoints	3	2013	2016	-0.3181	-1.025	0.0599	0.087
	NH AIAN	NH AIAN - 4 joinpoints	4	2016	2019	-1.8622*	-2.9203	-1.3836	< 0.001
	NH API	NH API - 3 joinpoints	1	2000	2005	-0.6841*	-1.7251	-0.2345	0.0060
	NH API	NH API - 3 joinpoints	2	2005	2011	0.3903*	0.0187	1.3363	0.043
	NH API	NH API - 3 joinpoints	3	2011	2019	-1.2277*	-1.5619	-0.9766	0.0048
	NH Black	NH Black - 1 joinpoint	1	2000	2019	-1.8571*	-1.9522	-1.7629	< 0.001
	Latino	Latino - 3 joinpoints	1	2000	2002	-1.007	-2.1481	0.0411	0.058
	Latino	Latino - 3 joinpoints	2	2002	2006	-2.4322*	-3.0921	-0.6331	< 0.001
	Latino	Latino - 3 joinpoints	3	2006	2019	-1.1438*	-1.9381	-0.7791	0.0016
	NH White	NH White - 1 joinpoint	1	2000	2019	-1.1875*	-1.2417	-1.1344	< 0.001
Tennessee	NH AIAN	NH AIAN - 3 joinpoints	1	2000	2007	-0.3438	-0.9924	0.4387	0.23
	NH AIAN	NH AIAN - 3 joinpoints	2	2007	2011	-3.3647*	-4.5293	-1.0351	0.040
	NH AIAN	NH AIAN - 3 joinpoints	3	2011	2019	-1.6949	-2.2269	0.1156	0.055
	NH API	NH API - 2 joinpoints	1	2000	2015	-0.769*	-0.8536	-0.6543	0.0048
	NH API	NH API - 2 joinpoints	2	2015	2019	-1.8572*	-2.9379	-1.312	< 0.001
	NH Black	NH Black - 4 joinpoints	1	2000	2002	-0.5624*	-1.0414	-0.1371	0.010
	NH Black	NH Black - 4 joinpoints	2	2002	2008	-1.3243*	-1.5709	-1.2257	< 0.001

	NH Black	NH Black - 4 joinpoints	3	2008	2014	-1.9842*	-2.14	-1.812	< 0.001
	NH Black	NH Black - 4 joinpoints	4	2014	2019	-2.4295*	-2.7394	-2.2832	< 0.001
	Latino	Latino - 2 joinpoints	1	2000	2007	-1.0824*	-1.4444	-0.8068	< 0.001
	Latino	Latino - 2 joinpoints	2	2007	2019	0.65*	0.5237	0.7965	< 0.001
	NH White	NH White - 2 joinpoints	1	2000	2015	-0.6924*	-0.7259	-0.656	< 0.001
	NH White	NH White - 2 joinpoints	2	2015	2019	-1.6866*	-1.9459	-1.4293	< 0.001
Texas	NH AIAN	NH AIAN - 3 joinpoints	1	2000	2005	1.9003*	1.1112	2.7862	0.014
	NH AIAN	NH AIAN - 3 joinpoints	2	2005	2016	-1.0251	-1.1838	1.8242	0.088
	NH AIAN	NH AIAN - 3 joinpoints	3	2016	2019	-2.2209*	-4.2019	-1.218	< 0.001
	NH API	NH API - 4 joinpoints	1	2000	2007	-1.391*	-2.206	-0.4729	0.030
	NH API	NH API - 4 joinpoints	2	2007	2010	-0.0632	-1.7278	0.4078	0.51
	NH API	NH API - 4 joinpoints	3	2010	2015	-1.0872*	-1.9573	-0.2088	0.030
	NH API	NH API - 4 joinpoints	4	2015	2019	-2.258*	-3.5088	-1.5973	< 0.001
	NH Black	NH Black - 3 joinpoints	1	2000	2002	-0.9632*	-1.4949	-0.5469	< 0.001
	NH Black	NH Black - 3 joinpoints	2	2002	2010	-1.801*	-1.9513	-1.7174	< 0.001
	NH Black	NH Black - 3 joinpoints	3	2010	2019	-2.2326*	-2.3382	-2.1601	< 0.001
	Latino	Latino - 3 joinpoints	1	2000	2007	-1.3574*	-1.9653	-1.1374	< 0.001
	Latino	Latino - 3 joinpoints	2	2007	2013	-0.456	-0.7621	0.269	0.15
	Latino	Latino - 3 joinpoints	3	2013	2019	-2.0105*	-2.3658	-1.7389	< 0.001
	NH White	NH White - 3 joinpoints	1	2000	2005	-1.2689*	-1.7391	-1.0719	< 0.001
	NH White	NH White - 3 joinpoints	2	2005	2015	-0.8346*	-0.904	-0.6573	< 0.001
	NH White	NH White - 3 joinpoints	3	2015	2019	-1.9925*	-2.2774	-1.7196	< 0.001

Utah	NH AIAN	NH AIAN - 2 joinpoints	1	2000	2006	0.1564	-0.3543	1.0399	0.50
	NH AIAN	NH AIAN - 2 joinpoints	2	2006	2019	-1.3903*	-1.6183	-1.2145	< 0.001
	NH API	NH API - 3 joinpoints	1	2000	2006	-1.4437*	-1.9024	-1.1955	< 0.001
	NH API	NH API - 3 joinpoints	2	2006	2015	-0.5736*	-0.7192	-0.2498	0.015
	NH API	NH API - 3 joinpoints	3	2015	2019	-1.7598*	-2.4315	-1.3359	< 0.001
	NH Black	NH Black - 2 joinpoints	1	2000	2006	-2.7818*	-3.4232	-2.478	< 0.001
	NH Black	NH Black - 2 joinpoints	2	2006	2019	-1.7185*	-1.8218	-1.5903	< 0.001
	Latino	Latino - 1 joinpoint	1	2000	2019	-1.3932*	-1.4792	-1.3079	< 0.001
	NH White	NH White - 2 joinpoints	1	2000	2016	-1.051*	-1.0802	-1.0147	< 0.001
	NH White	NH White - 2 joinpoints	2	2016	2019	-1.67*	-2.2081	-1.359	< 0.001
Vermont	NH AIAN	NH AIAN - 4 joinpoints	1	2000	2007	0.2417	-0.2015	0.6486	0.15
	NH AIAN	NH AIAN - 4 joinpoints	2	2007	2012	-3.4714*	-4.365	-0.1625	0.046
	NH AIAN	NH AIAN - 4 joinpoints	3	2012	2016	-1.1585*	-3.3828	-0.2116	0.0084
	NH AIAN	NH AIAN - 4 joinpoints	4	2016	2019	-3.0472*	-4.5112	-1.9989	< 0.001
	NH API	NH API - 3 joinpoints	1	2000	2007	-0.6477*	-1.6358	-0.3181	< 0.001
	NH API	NH API - 3 joinpoints	2	2007	2013	0.6645*	0.1844	1.7108	0.0032
	NH API	NH API - 3 joinpoints	3	2013	2019	-2.3034*	-2.7731	-1.8617	< 0.001
	NH Black	NH Black - 3 joinpoints	1	2000	2005	-3.2276*	-4.3362	-2.734	< 0.001
	NH Black	NH Black - 3 joinpoints	2	2005	2013	-1.8899*	-2.6106	-1.591	< 0.001
	NH Black	NH Black - 3 joinpoints	3	2013	2019	-1.1881*	-1.5485	-0.1232	0.031
	Latino	Latino - 4 joinpoints	1	2000	2002	0.0482	-1.2773	1.2744	0.88
	Latino	Latino - 4 joinpoints	2	2002	2007	-1.5988	-2.5657	0.4125	0.099

	Latino	Latino - 4 joinpoints	3	2007	2014	0.2316	-1.0195	1.0602	0.20
	Latino	Latino - 4 joinpoints	4	2014	2019	-1.1558*	-1.8965	-0.5853	0.017
	NH White	NH White - 3 joinpoints	1	2000	2006	-1.5973*	-1.92	-1.4299	< 0.001
	NH White	NH White - 3 joinpoints	2	2006	2015	-0.9825*	-1.0817	-0.8036	< 0.001
	NH White	NH White - 3 joinpoints	3	2015	2019	-1.9916*	-2.3709	-1.7247	< 0.001
Virginia	NH AIAN	NH AIAN - 2 joinpoints	1	2000	2005	-0.3639	-0.9758	0.8912	0.37
	NH AIAN	NH AIAN - 2 joinpoints	2	2005	2019	-1.6636*	-1.8651	-1.5182	< 0.001
	NH API	NH API - 2 joinpoints	1	2000	2015	-1.6249*	-1.6991	-1.4987	0.0040
	NH API	NH API - 2 joinpoints	2	2015	2019	-2.4373*	-3.5418	-1.9384	< 0.001
	NH Black	NH Black - 2 joinpoints	1	2000	2002	-1.5319*	-2.0071	-1.2096	< 0.001
	NH Black	NH Black - 2 joinpoints	2	2002	2019	-2.2149*	-2.2503	-2.1918	< 0.001
	Latino	Latino - 3 joinpoints	1	2000	2002	0.5687	-0.4721	1.3896	0.32
	Latino	Latino - 3 joinpoints	2	2002	2017	-1.1007*	-1.1943	-0.9871	< 0.001
	Latino	Latino - 3 joinpoints	3	2017	2019	-2.17*	-3.0146	-1.2233	< 0.001
	NH White	NH White - 2 joinpoints	1	2000	2016	-1.335*	-1.3739	-1.2695	< 0.001
	NH White	NH White - 2 joinpoints	2	2016	2019	-1.9569*	-2.849	-1.5281	< 0.001
Washington	NH AIAN	NH AIAN - 3 joinpoints	1	2000	2006	0.3221	-0.2197	1.0679	0.15
	NH AIAN	NH AIAN - 3 joinpoints	2	2006	2016	-1.5111*	-1.7114	-0.1606	0.044
	NH AIAN	NH AIAN - 3 joinpoints	3	2016	2019	-2.9169*	-4.9118	-1.8126	< 0.001
	NH API	NH API - 2 joinpoints	1	2000	2015	-1.3394*	-1.4136	-1.2576	< 0.001
	NH API	NH API - 2 joinpoints	2	2015	2019	-2.7334*	-3.5173	-2.2624	< 0.001
	NH Black	NH Black - 3 joinpoints	1	2000	2002	-0.726*	-1.3759	-0.1934	0.012

	NH Black	NH Black - 3 joinpoints	2	2002	2013	-1.9344*	-2.0185	-1.8665	< 0.001
	NH Black	NH Black - 3 joinpoints	3	2013	2019	-2.7527*	-2.978	-2.5788	< 0.001
	Latino	Latino - 3 joinpoints	1	2000	2007	-1.3315*	-1.798	-1.0689	0.0016
	Latino	Latino - 3 joinpoints	2	2007	2014	-0.431	-0.9272	0.249	0.20
	Latino	Latino - 3 joinpoints	3	2014	2019	-0.9947*	-1.8428	-0.6679	< 0.001
	NH White	NH White - 3 joinpoints	1	2000	2002	-0.6845*	-1.1356	-0.2969	< 0.001
	NH White	NH White - 3 joinpoints	2	2002	2015	-1.308*	-1.3642	-1.2696	< 0.001
	NH White	NH White - 3 joinpoints	3	2015	2019	-1.8236*	-2.1877	-1.6291	< 0.001
West Virginia	NH AIAN	NH AIAN - 2 joinpoints	1	2000	2005	0.3642	-0.373	1.7109	0.30
	NH AIAN	NH AIAN - 2 joinpoints	2	2005	2019	-1.3554*	-1.5942	-1.1829	< 0.001
	NH API	NH API - 4 joinpoints	1	2000	2006	-0.3146	-1.1896	0.7638	0.13
	NH API	NH API - 4 joinpoints	2	2006	2012	0.6048	-0.6887	1.4101	0.17
	NH API	NH API - 4 joinpoints	3	2012	2015	-0.1424	-1.5464	0.956	0.86
	NH API	NH API - 4 joinpoints	4	2015	2019	-1.3529*	-2.513	-0.5818	0.0056
	NH Black	NH Black - 2 joinpoints	1	2000	2013	-1.1891*	-1.252	-1.1184	< 0.001
	NH Black	NH Black - 2 joinpoints	2	2013	2019	-2.0762*	-2.3333	-1.8779	< 0.001
	Latino	Latino - 4 joinpoints	1	2000	2002	-1.0957*	-2.0599	-0.2594	0.0052
	Latino	Latino - 4 joinpoints	2	2002	2006	-2.8459*	-3.4371	-2.3808	0.0012
	Latino	Latino - 4 joinpoints	3	2006	2010	-1.4041*	-2.0181	-0.4133	0.024
	Latino	Latino - 4 joinpoints	4	2010	2019	-0.2441	-0.4154	0.0844	0.080
	NH White	NH White - 2 joinpoints	1	2000	2014	-0.7793*	-0.8256	-0.7322	< 0.001
	NH White	NH White - 2 joinpoints	2	2014	2019	-1.9009*	-2.1367	-1.6755	< 0.001

Wisconsin	NH AIAN	NH AIAN - 2 joinpoints	1	2000	2006	0.4211	-0.0588	1.0647	0.094
	NH AIAN	NH AIAN - 2 joinpoints	2	2006	2019	-1.773*	-1.9617	-1.6117	< 0.001
	NH API	NH API - 4 joinpoints	1	2000	2007	-1.0741*	-1.3946	-0.8645	0.0028
	NH API	NH API - 4 joinpoints	2	2007	2011	0.7804*	0.2529	1.4363	0.038
	NH API	NH API - 4 joinpoints	3	2011	2015	-0.6559*	-1.2148	-0.0843	0.041
	NH API	NH API - 4 joinpoints	4	2015	2019	-2.2741*	-2.9847	-1.8888	< 0.001
	NH Black	NH Black - 4 joinpoints	1	2000	2002	-0.526*	-0.9451	-0.1455	0.0012
	NH Black	NH Black - 4 joinpoints	2	2002	2006	-1.453*	-1.7246	-1.2696	< 0.001
	NH Black	NH Black - 4 joinpoints	3	2006	2012	-0.8956*	-1.0151	-0.6166	< 0.001
	NH Black	NH Black - 4 joinpoints	4	2012	2019	-1.4703*	-1.586	-1.3751	< 0.001
	Latino	Latino - 2 joinpoints	1	2000	2016	0.1423*	0.0609	0.2781	0.0016
	Latino	Latino - 2 joinpoints	2	2016	2019	-1.0888*	-2.6075	-0.2412	0.0028
	NH White	NH White - 2 joinpoints	1	2000	2015	-1.049*	-1.0835	-1.0109	< 0.001
	NH White	NH White - 2 joinpoints	2	2015	2019	-2.166*	-2.3808	-1.8935	< 0.001
Wyoming	NH AIAN	NH AIAN - 2 joinpoints	1	2000	2005	-0.5975	-1.2229	1.01	0.23
	NH AIAN	NH AIAN - 2 joinpoints	2	2005	2019	-1.5462*	-1.9617	-1.4104	< 0.001
	NH API	NH API - 3 joinpoints	1	2000	2007	-1.7937*	-2.907	-1.3912	< 0.001
	NH API	NH API - 3 joinpoints	2	2007	2015	-0.5615	-0.8887	0.6714	0.22
	NH API	NH API - 3 joinpoints	3	2015	2019	-2.4539*	-4.0261	-1.6605	< 0.001
	NH Black	NH Black - 3 joinpoints	1	2000	2007	-3.2688*	-3.785	-3.0183	< 0.001
	NH Black	NH Black - 3 joinpoints	2	2007	2010	-1.0954*	-2.0013	-0.6204	< 0.001
	NH Black	NH Black - 3 joinpoints	3	2010	2019	-2.5738*	-2.8691	-2.4317	< 0.001

Latino	Latino - 2 joinpoints	1	2000	2007	-2.1476*	-2.8504	-1.8372	< 0.001
Latino	Latino - 2 joinpoints	2	2007	2019	-1.1843*	-1.3315	-0.9484	< 0.001
NH White	NH White - 2 joinpoints	1	2000	2016	-1.5017*	-1.5388	-1.4141	< 0.001
NH White	NH White - 2 joinpoints	2	2016	2019	-2.0259*	-2.8339	-1.6183	< 0.001

APC, annual percentage change; CI, confidence interval; NH, non-Hispanic; AIAN, American Indian or Alaska Native; API, Asian or Pacific Islander

Table S3. Top 10 counties with highest and lowest age-standardized neoplasm-related mortality rates for each race/ethnicity in 2019.

	State	County	Age-standardized mortality rate (95% UI)					
**Rank**	**NH AIAN**							
1	Maine	Cumberland County	559	(	316	–	871	)
2	New Jersey	Cumberland County	541	(	324	–	825	)
3	Kansas	Sedgwick County	502	(	396	–	642	)
4	Maine	Washington County	495	(	294	–	764	)
5	Maine	Penobscot County	474	(	289	–	721	)
6	South Dakota	Minnehaha County	463	(	348	–	598	)
7	Montana	Cascade County	463	(	360	–	571	)
8	Mississippi	Neshoba County	447	(	356	–	555	)
9	Kansas	Shawnee County	444	(	342	–	564	)
10	North Carolina	Cumberland County	431	(	337	–	540	)
…								
498	Missouri	Saint Louis County	78	(	58	–	105	)
499	Texas	Nueces County	77	(	52	–	109	)
500	New York	Kings County	77	(	47	–	122	)
501	Ohio	Montgomery County	76	(	57	–	103	)
502	Texas	Travis County	72	(	53	–	97	)
503	Ohio	Hamilton County	72	(	54	–	93	)
504	Indiana	Marion County	69	(	52	–	90	)
505	New York	Queens County	68	(	41	–	105	)
506	Alabama	Madison County	60	(	44	–	79	)

507	Alabama	Lawrence County	59	(	42	–	80	)
	**NH Asian Pacific Islanders**							
1	Minnesota	Nobles County	199	(	151	–	261	)
2	Kansas	Geary County	198	(	151	–	253	)
3	Kansas	Wyandotte County	197	(	157	–	248	)
4	Mississippi	Harrison County	191	(	157	–	227	)
5	Oklahoma	Garfield County	186	(	148	–	230	)
6	Louisiana	Iberia Parish	185	(	144	–	230	)
7	Iowa	Buena Vista County	181	(	137	–	234	)
8	Minnesota	Ramsey County	178	(	156	–	204	)
9	Iowa	Polk County	178	(	151	–	211	)
10	Wisconsin	Sheboygan County	177	(	142	–	216	)
…								
679	Alaska	Ketchikan Gateway Borough	72	(	49	–	106	)
679	Alaska	Petersburg Borough	72	(	49	–	106	)
679	Alaska	Prince of Wales–Hyder Census Area	72	(	49	–	106	)
679	Alaska	Prince of Wales-Outer Ketchikan Census Area	72	(	49	–	106	)
679	Alaska	Wrangell City and Borough	72	(	49	–	106	)
679	Alaska	Wrangell-Petersburg Census Area	72	(	49	–	106	)
685	Michigan	Washtenaw County	72	(	61	–	84	)
686	Indiana	Tippecanoe County	71	(	56	–	90	)
687	Michigan	Houghton County	68	(	47	–	95	)
688	Virginia	Montgomery County	64	(	48	–	82	)
689	New Mexico	Los Alamos County	60	(	43	–	82	)
690	Alaska	Aleutian Islands Census Area	35	(	20	–	57	)
690	Alaska	Aleutians East Borough	35	(	20	–	57	)

690	Alaska	Aleutians West Census Area	35	(	20	–	57	)
	**NH Black**							
1	Florida	Union County	535	(	450	–	627	)
2	Virginia	Martinsville City	431	(	361	–	505	)
3	Louisiana	East Carroll Parish	358	(	318	–	406	)
4	Texas	Anderson County	354	(	317	–	398	)
5	Mississippi	Madison County	351	(	325	–	379	)
6	Mississippi	Grenada County	343	(	307	–	381	)
7	Georgia	Miller County	340	(	283	–	404	)
8	Mississippi	Bolivar County	338	(	311	–	368	)
9	Virginia	Colonial Heights City	336	(	260	–	427	)
10	Louisiana	Madison Parish	335	(	297	–	378	)
…								
1506	Minnesota	Steele County	121	(	89	–	159	)
1507	South Dakota	Pennington County	120	(	93	–	151	)
1508	Minnesota	Kandiyohi County	119	(	88	–	159	)
1509	California	Lassen County	119	(	89	–	157	)
1510	New Mexico	Santa Fe County	119	(	95	–	145	)
1511	Washington	Whitman County	114	(	89	–	141	)
1512	Minnesota	Rice County	111	(	87	–	139	)
1513	North Dakota	Burleigh County	102	(	74	–	135	)
1514	North Dakota	Ward County	102	(	72	–	142	)
1515	Michigan	Chippewa County	69	(	47	–	100	)
	**Latino or Hispanic**							
1	Texas	Anderson County	402	(	345	–	468	)
2	Hawaii	Honolulu County	250	(	223	–	279	)

3	Hawaii	Hawaii County	235	(	201	–	275	)
4	Texas	Martin County	220	(	178	–	275	)
5	Colorado	Huerfano County	219	(	187	–	256	)
6	Texas	Dawson County	218	(	189	–	251	)
7	Texas	San Patricio County	217	(	197	–	237	)
8	Texas	Kleberg County	216	(	195	–	238	)
9	Texas	Jim Hogg County	215	(	188	–	244	)
10	Texas	Karnes County	214	(	189	–	243	)
…								
1494	Mississippi	Yazoo County	61	(	47	–	79	)
1495	Ohio	Athens County	60	(	45	–	77	)
1496	Mississippi	Rankin County	60	(	46	–	76	)
1497	Mississippi	Pontotoc County	60	(	44	–	78	)
1498	Mississippi	Adams County	59	(	42	–	78	)
1499	West Virginia	Monongalia County	59	(	44	–	74	)
1500	Georgia	Telfair County	57	(	43	–	75	)
1501	Alaska	Aleutian Islands Census Area	36	(	17	–	72	)
1501	Alaska	Aleutians East Borough	36	(	17	–	72	)
1501	Alaska	Aleutians West Census Area	36	(	17	–	72	)
	**NH White**							
1	Florida	Union County	452	(	413	–	495	)
2	Virginia	Petersburg City	438	(	384	–	496	)
3	Virginia	Galax City	372	(	329	–	419	)
4	Virginia	Martinsville City	354	(	303	–	408	)
5	Kentucky	Perry County	341	(	320	–	364	)
6	Virginia	Hopewell City	332	(	297	–	369	)

7	Kentucky	Powell County	325	(	297	–	353	)
8	Kentucky	Clay County	321	(	298	–	346	)
9	Virginia	Covington City	315	(	266	–	370	)
10	South Carolina	Marlboro County	313	(	286	–	341	)
…								
3087	Wyoming	Teton County	109	(	99	–	121	)
3088	Colorado	San Miguel County	103	(	88	–	120	)
3089	Alaska	Aleutian Islands Census Area	95	(	58	–	149	)
3089	Alaska	Aleutians East Borough	95	(	58	–	149	)
3089	Alaska	Aleutians West Census Area	95	(	58	–	149	)
3092	Texas	Starr County	94	(	76	–	115	)
3093	Colorado	Eagle County	86	(	77	–	95	)
3094	Colorado	Pitkin County	79	(	70	–	88	)
3095	Colorado	Summit County	69	(	60	–	77	)
3096	Alaska	North Slope Borough	59	(	34	–	97	)

Abbreviations; AIAN, American Indian or Alaska Native; NH, non-Hispanic; UI, uncertainty interval.

Table S4. County-level correlation coefficients between socioeconomic status variables and age-standardized neoplasm-related mortality rates for each race/ethnicity.

Variables	Race	2000 Value (95% CI)	2010 Value (95% CI)	2019 Value (95% CI)
Household income	NH AIAN	-0.076 (-0.163 - 0.013)	-0.183 (-0.267 - -0.097)	-0.239 (-0.321 - -0.154)
	NH API	-0.002 (-0.076 - 0.073)	-0.050 (-0.125 - 0.024)	-0.179 (-0.250 - -0.105)
	NH Black	-0.333 (-0.378 - -0.288)	-0.477 (-0.515 - -0.437)	-0.529 (-0.565 - -0.492)
	Latino	-0.058 (-0.108 - -0.007)	0.004 (-0.047 - 0.054)	-0.048 (-0.098 - 0.003)
	Total	-0.309 (-0.340 - -0.277)	-0.512 (-0.537 - -0.485)	-0.590 (-0.612 - -0.566)
	NH White	-0.260 (-0.293 - -0.227)	-0.472 (-0.499 - -0.444)	-0.550 (-0.574 - -0.524)
Poverty rate	NH AIAN	-0.003 (-0.091 - 0.086)	0.064 (-0.024 - 0.152)	0.159 (0.072 - 0.244)
	NH API	0.077 (0.002 - 0.151)	0.051 (-0.023 - 0.126)	0.082 (0.008 - 0.156)
	NH Black	0.350 (0.305 - 0.393)	0.459 (0.418 - 0.498)	0.509 (0.471 - 0.545)
	Latino	0.130 (0.080 - 0.179)	0.028 (-0.023 - 0.079)	0.086 (0.036 - 0.136)
	Total	0.402 (0.372 - 0.431)	0.545 (0.520 - 0.569)	0.570 (0.546 - 0.594)
	NH White	0.336 (0.304 - 0.366)	0.491 (0.464 - 0.518)	0.520 (0.494 - 0.545)

CI, confidence interval; NH, non-Hispanic; AIAN, American Indian or Alaska Native; API, Asian or Pacific Islander

Figure S1. Trends in age-standardized neoplasm-related mortality rates by US state and racial/ethnic group, stratified by sex.

(a) Men

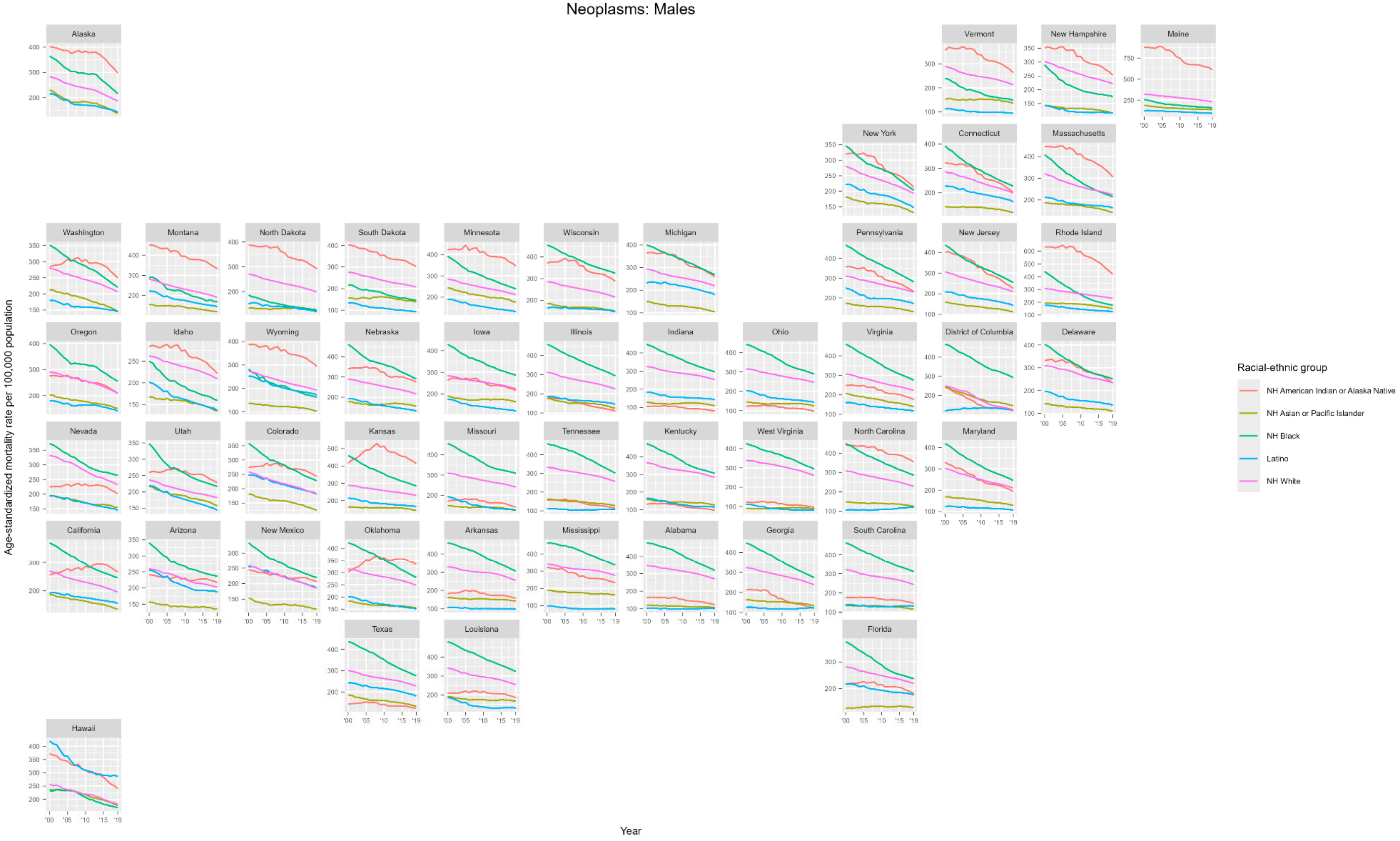

(b) Women

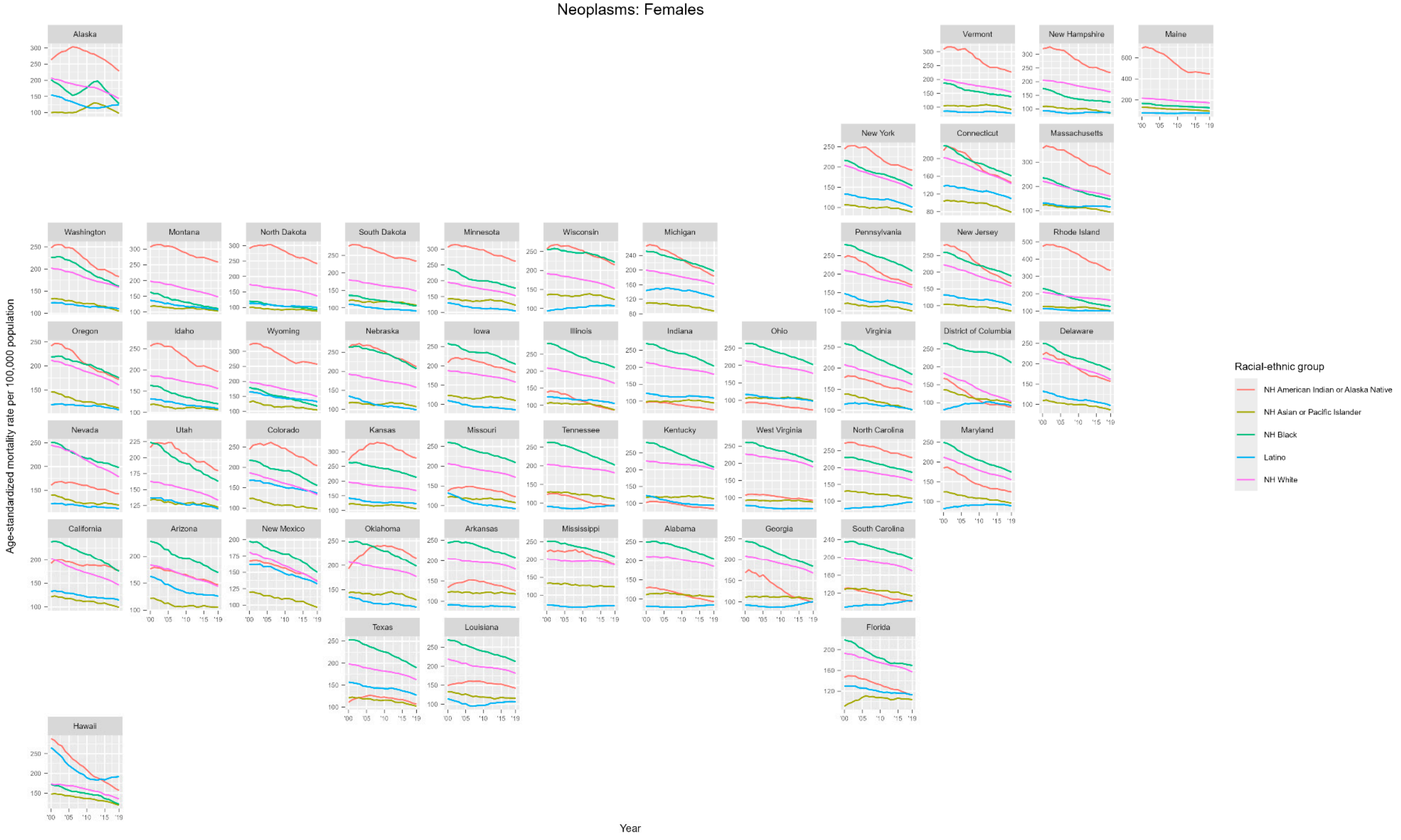
Neoplasms: Females
Age-standardized mortality rate per 100,000 population
Year
Alaska
Vermont
New Hampshire
Maine
New York
Connecticut
Massachusetts
Washington
Montana
North Dakota
South Dakota
Minnesota
Wisconsin
Michigan
Pennsylvania
New Jersey
Rhode Island
Oregon
Idaho
Wyoming
Nebraska
Iowa
Illinois
Indiana
Ohio
Virginia
District of Columbia
Delaware
Nevada
Utah
Colorado
Kansas
Missouri
Tennessee
Kentucky
West Virginia
North Carolina
Maryland
California
Arizona
New Mexico
Oklahoma
Arkansas
Mississippi
Alabama
Georgia
South Carolina
Texas
Louisiana
Florida
Hawaii
Racial-ethnic group
NH American Indian or Alaska Native
NH Asian or Pacific Islander
NH Black
Latino
NH White

(c) Combined

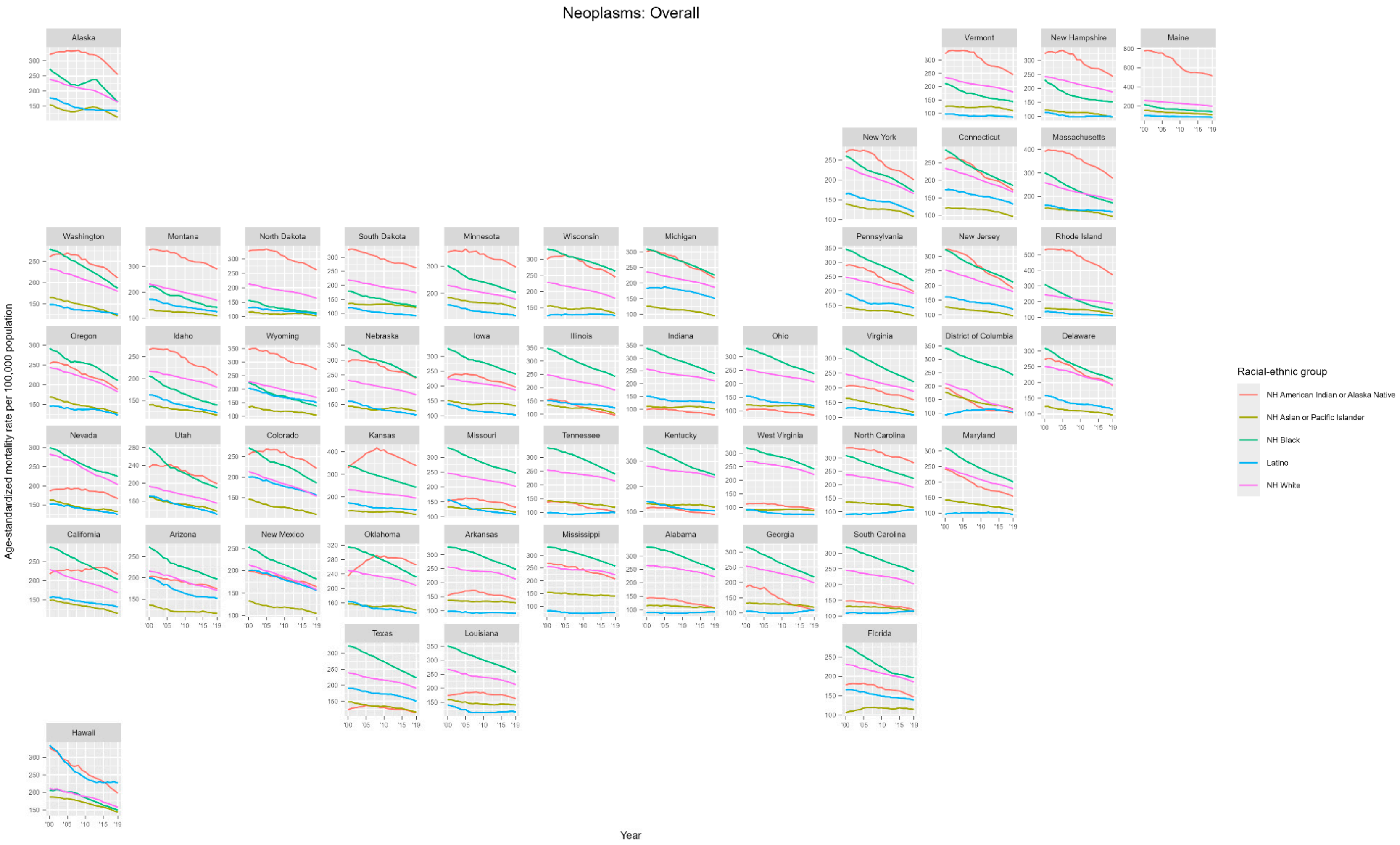
Neoplasms: Overall
Age-standardized mortality rate per 100,000 population
Year
Alaska
Vermont
New Hampshire
Maine
New York
Connecticut
Massachusetts
Washington
Montana
North Dakota
South Dakota
Minnesota
Wisconsin
Michigan
Pennsylvania
New Jersey
Rhode Island
Oregon
Idaho
Wyoming
Nebraska
Iowa
Illinois
Indiana
Ohio
Virginia
District of Columbia
Delaware
Nevada
Utah
Colorado
Kansas
Missouri
Tennessee
Kentucky
West Virginia
North Carolina
Maryland
California
Arizona
New Mexico
Oklahoma
Arkansas
Mississippi
Alabama
Georgia
South Carolina
Texas
Louisiana
Florida
Hawaii
Racial-ethnic group
NH American Indian or Alaska Native
NH Asian or Pacific Islander
NH Black
Latino
NH White

Figure S2. Absolute change in age-standardized neoplasm-related mortality rates between 2000 and 2019 by US state and racial/ethnic group.

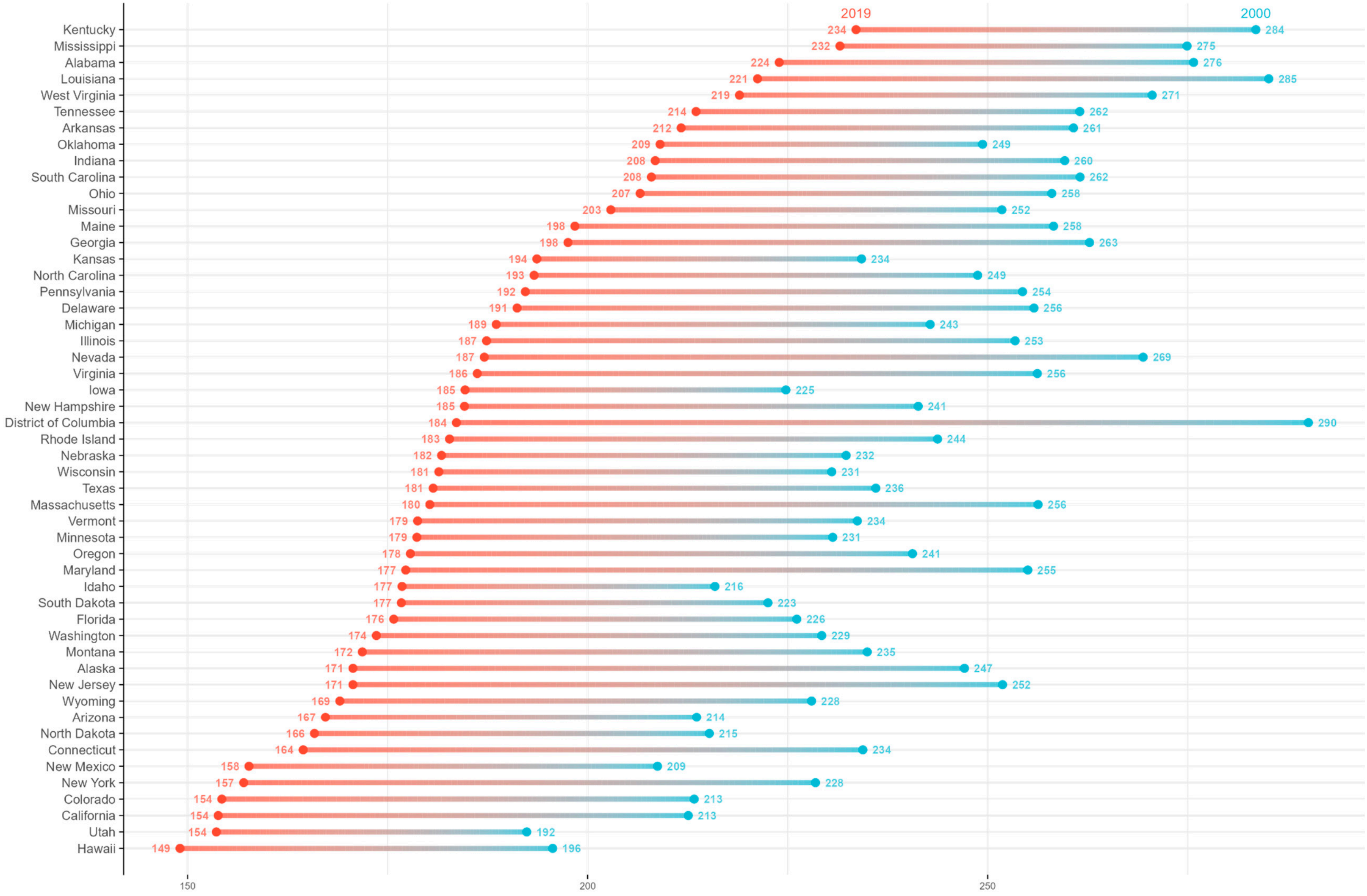

Figure S3. County-level correlation coefficients between preventative measures and age-standardized neoplasm-related mortality rates for each race/ethnicity (2011 vs. 2019).

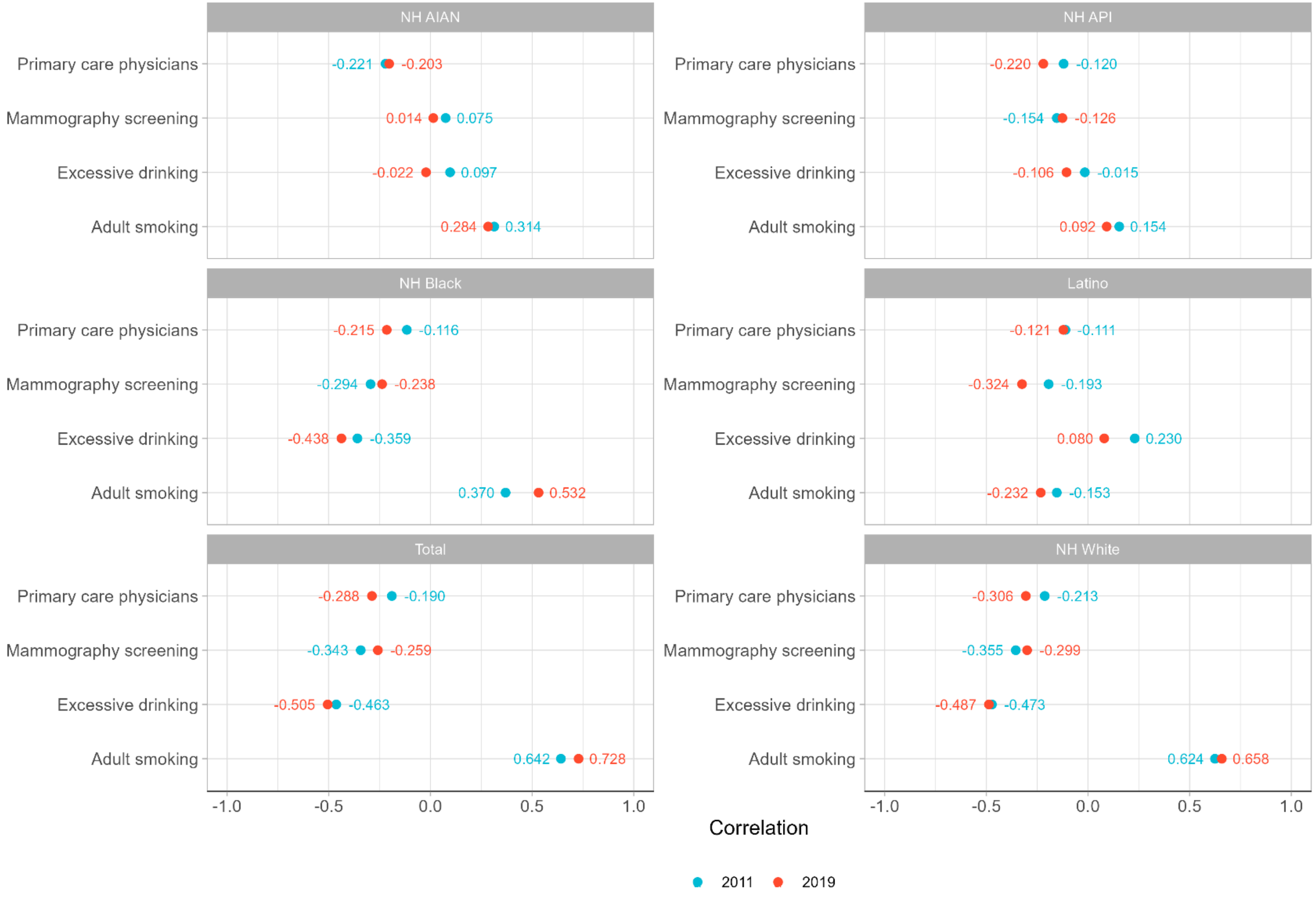

Abbreviations: NH, non-Hispanic; AIAN, American Indian or Alaska Native; API, Asian or Pacific Islander
